# Chronic arsenic trioxide exposure leads to enhanced aggressiveness via Met oncogene addiction in cancer cells

**DOI:** 10.18632/oncotarget.8415

**Published:** 2016-03-28

**Authors:** Kushtrim Kryeziu, Christine Pirker, Bernhard Englinger, Sushilla van Schoonhoven, Melanie Spitzwieser, Thomas Mohr, Wilfried Körner, Regina Weinmüllner, Koray Tav, Johannes Grillari, Margit Cichna-Markl, Walter Berger, Petra Heffeter

**Affiliations:** ^1^ Department of Medicine I, Institute of Cancer Research and Comprehensive Cancer Center, Medical University Vienna, Vienna, Austria; ^2^ Research Platform “Translational Cancer Therapy Research”, Vienna, Austria; ^3^ Department of Analytical Chemistry, University of Vienna, Vienna, Austria; ^4^ Department of Environmental Geosciences, University of Vienna, Vienna, Austria; ^5^ Christian Doppler Laboratory on Biotechnology of Skin Aging, Department of Biotechnology, University of Natural Resources and Applied Life Sciences, Vienna, Austria; ^6^ Evercyte GmbH, Vienna, Austria

**Keywords:** arsenic trioxide, carcinogen, aggressiveness, resistance, Met

## Abstract

As an environmental poison, arsenic is responsible for many cancer deaths. Paradoxically, arsenic trioxide (ATO) presents also a powerful therapy used to treat refractory acute promyelocytic leukemia (APL) and is intensively investigated for treatment of other cancer types. Noteworthy, cancer therapy is frequently hampered by drug resistance, which is also often associated with enhancement of tumor aggressiveness.

In this study, we analyzed ATO-selected cancer cells (A2780_ATO_) for the mechanisms underlying their enhanced tumorigenicity and aggressiveness. These cells were characterized by enhanced proliferation and spheroid growth as well as increased tumorigenicity of xenografts in SCID mice. Noteworthy, subsequent studies revealed that overexpression of Met receptor was the underlying oncogenic driver of these effects, as A2780_ATO_ cells were characterized by collateral sensitivity against Met inhibitors. This finding was also confirmed by array comparative genomic hybridization (array CGH) and whole genome gene expression arrays, which revealed that Met overexpression by chronic ATO exposure was based on the transcriptional regulation via activation of AP-1. Finally, it was shown that treatment with the Met inhibitor crizotinib was also effective against A2780_ATO_ cell xenografts *in vivo*, indicating that targeting of Met presents a promising strategy for the treatment of Met-overexpressing tumors after either arsenic exposure or failure to ATO treatment.

## INTRODUCTION

Arsenic is known for its therapeutic potential and has been used for more than two millennia in the treatment of various diseases including cancer [1]. In the late 1970s, ATO was rediscovered as a potent anticancer agent against acute promyelocytic leukemia (APL) and finally approved by the U.S. Food and Drug Administration (FDA) and European Medicines Agency (EMA) in 2000 and 2002, respectively (www.fda.gov and www.ema.europa.eu). Next to platinum-based drugs, ATO became the only clinically used metal-based anticancer drug [2].

In APL, ATO at low doses induced cell differentiation by targeting the PML-RARα fusion oncoprotein, whereas at higher doses it induced apoptosis [3]. Other mechanisms of action ascribed to ATO are the induction of reactive oxygen species (ROS), decrease of the mitochondrial membrane potential, down-regulation of anti-apoptotic proteins or activation of proto-oncogenes [4, 5]. Auspicious results from preclinical studies motivated several clinical trials in solid tumors, unfortunately without significant efficacy [6–8]. Nevertheless, ATO as a single treatment or in combination with other drugs is currently under investigation in a variety of solid cancers including lung, bladder, liver, colon, brain, breast, cervix, esophagus, and skin cancer (www.clinicaltrials.gov). Like for many other anticancer drugs, limitations of ATO are based on intrinsic and acquired drug resistance [2]. Noteworthy, development of drug resistance is frequently associated with the appearance of a more aggressive cancer phenotype [9, 10]. Consequently, it is of interest to investigate the underlying mechanisms of therapy failure and to use this knowledge for further treatment strategies.

Arsenic is not only in the focus of interest due to its anticancer activity but, besides, it is a primary concern as environmental poison with 200 million people estimated to be at risk of toxic exposure worldwide [11]. Especially Bangladesh, India, Vietnam, Thailand, Mexico, but also the United States are among the countries that reported poisoning from elevated levels of arsenic in groundwater and soil [11, 12]. First signs of chronic arsenic poisoning (arsenicosis) are cutaneous manifestations including melanosis, keratosis, and skin cancers [13]. Furthermore, long-term exposure to arsenic was shown to cause cancers of the bladder, lung, and liver [14]. However, although some mechanisms underlying this carcinogenicity have already been described, the exact mode of action is still a matter of discussion. Arsenic and its metabolites have been shown to generate ROS as a potential inducer of genomic instability through DNA damage, impaired DNA repair or telomere dysfunction [15]. Mutations originating from this DNA damage might silence tumor suppressors like pro-apoptotic genes or activate proto-oncogenes which in turn lead to genomic instability and cellular transformation [15]. As an example, the epidermal growth factor receptor (EGFR) has been described as one of the potential oncogenes to be activated directly or via SRC proto-oncogene in keratinocytes and human lung cells after chronic arsenic exposure [16–18].

Recently, we revealed that solid cancer cells manage to escape from ATO treatment by stimulation of DNA damage repair via the EGFR signaling pathway [19]. In order to investigate the potential anticancer activity of arsenic in solid tumors, cell-transforming capabilities of ATO have to be considered as well. Thus, in this study, we aimed to investigate the impact of acquired ATO resistance on the aggressiveness of an ATO-sensitive ovarian cancer cell model and elucidate the underlying molecular mechanisms.

## RESULTS

### Activity of ATO against various solid cancer cell lines and selection for acquired resistance

A panel of different well-established human cell models from various solid tumors was used in this study. Overall, ATO exhibited an anticancer activity in the lower μM range (Supplementary Figure S1A). Accordingly, environmentally and clinically relevant concentrations of arsenic range from 100 pmol/L – 2 μmol/L [3, 20]. In our test panel, the ovarian cancer cell model A2780 was the most sensitive with an IC_50_ of 1.2 μmol/L. Thus, this cell line was selected to generate an ATO-resistant subline and to analyze the response mechanisms to chronic ATO exposure.

**Figure 1:**
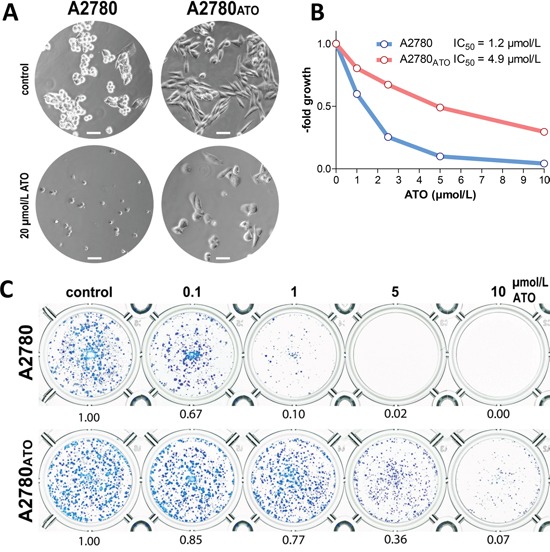
Morphology and impact of ATO on cell viability of A2780 and A2780_ATO_ **A.** Photomicrographs (phase contrast) of untreated A2780 cells and the ATO-resistant subline in cell culture and response to 20 μmol/L ATO after 24 h exposure, respectively (scale bar = 50 μm). **B.** Viability of A2780 cells and the ATO-resistant subline after 72 h exposure with the indicated concentrations of ATO were analyzed with MTT assay. **C.** Long-term exposure of ATO (7 days of exposure) on cell viability of A2780 and A2780_ATO_ were analyzed with crystal violet assay. ATO activity is presented as relative effect normalized at the untreated control and is shown below each well.

The A2780_ATO_ subline selected for acquired ATO resistance showed an elongated mesenchymal-like cell shape in cell culture fostered by chronic ATO exposure compared to epitheloid parental A2780 cells (Figure 1A upper panel). In terms of ATO responsiveness, the A2780_ATO_ subline in comparison to parental A2780 cells was distinctly less responsive to 20 μmol/L ATO after 24 h (Figure 1A lower panel), showed a 4-fold resistance after three days in viability testing (Figure 1B), and up to 28-fold resistance after seven days of ATO treatment in clonogenicity assays (Figure 1C).

### Proliferation and tumor aggressiveness of ATO-resistant cells

**Figure 2:**
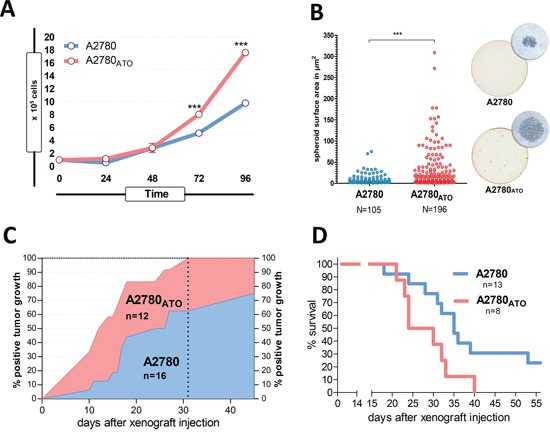
Proliferation, spheroid formation and tumorigenicity of ATO-resistant cells **A.** Proliferation rate of A2780 and A2780_ATO_ cells *in vitro* was measured by evaluation of cell viability at the indicated time-points (hours) using MTT assay, *** p < 0.001 analyzed with two-way ANOVA, Bonferroni post-test. **B.** Spheroid formation capacity of A2780 and A2780_ATO_ was analyzed using soft agar assay. Scatter plot diagram (left panel) shows counted spheroids and their size (area calculated as A=a×b×π; a=length/2 and b=width/2), *** p < 0.0001 analyzed with unpaired Welch-corrected t-test. A representative pair of spheroid growth in 12-wells and the respective micrographs is shown (20x objective magnification) (right panel). **C.** Tumorigenicity of A2780_ATO_ in comparison to the parental cells *in vivo*. Both cell lines were injected subcutaneously into SCID mice and were scored as “positive” from the day tumor diameters were measurable with a caliper. The vertical dotted line indicates the time when 100% of A2780_ATO_ xenografts were positive. **D.** Survival of SCID mice xenografted with A2780 cells and the ATO-resistant subline. Mice were sacrificed when the tumor reached more than 20 mm in one direction or upon tumor ulceration.

Noteworthy, the proliferation rate between A2780_ATO_ and A2780 was similar during the first 48 h after seeding. However, after 72 h, ATO-resistant cells proliferated significantly faster than the parental cells (up to 1.8-fold on day 4) (Figure 2A). Furthermore, soft agar assays showed enhanced three-dimensional growth of A2780_ATO_ cells indicated by both higher number as well as increased size of spheroid clones (Figure 2B). Faster proliferation and favorable spheroid growth were previously described as attributes of cancer cell aggressiveness and stemness [21, 22]. Consequently, to investigate the *in vivo* aggressiveness and tumorigenicity, both resistant and sensitive A2780 cells were analyzed as xenografts in SCID mice. Here, the tumorigenicity of A2780_ATO_ was distinctly higher with 100% tumor take after 31 days of xenotransplantation in comparison to 62% in the A2780 group. Moreover, A2780_ATO_ xenograft tumors appeared earlier (Figure 2C). Enhanced aggressiveness was also reflected by decreased survival of A2780_ATO_ tumor-bearing mice. Half-mean survival (50% of the investigated mice still alive) after xenotransplantation with A2780 was 35 days, whereas that of mice transplanted with A2780_ATO_ was distinctly shorter with only 25 days (Figure 2D). Altogether, these data indicate that ATO-resistant cells are more proliferative and aggressive as compared to the parental cells.

### Met receptor as a proliferative and survival factor in ATO-resistant cells

**Figure 3:**
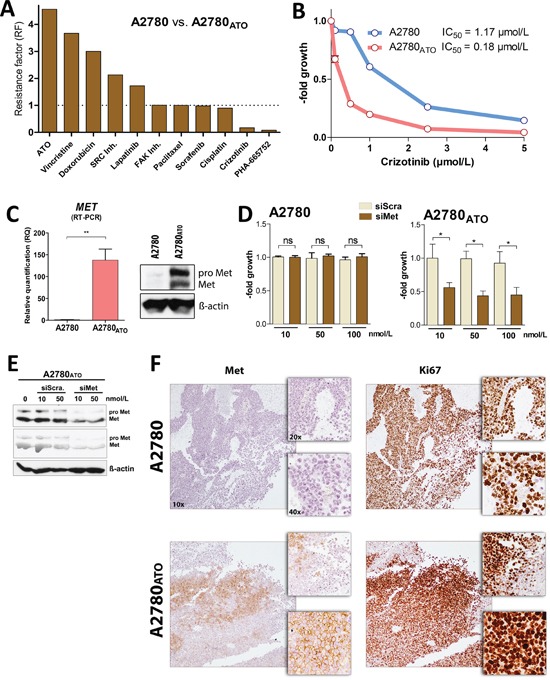
Met receptor as oncogenic driver of cell survival and proliferation **A.** Cross-resistance of various anticancer drugs in comparison to ATO shown as Resistance Factors (RF). RFs were calculated from IC_50_ of a drug for A2780_ATO_ divided by the one for A2780 cells, <1 - the given drug is more active in the resistant subclone than the parental cell line, = 1 - the drug is equally active in the resistant and parental cells and >1 - the given drug is more active in the parental cell line as compared to the resistant subline. **B.** Viability of A2780 and the ATO-resistant subline analyzed with MTT assay after 72 h exposure with the indicated concentrations of crizotinib. **C.** Left panel - *MET* mRNA expression analyzed with RT-PCR (normalized to *ACTB* gene expression, t-test, ** p < 0.01). Right panel - Met protein expression (pro Met – single-chain precursor and Met – matured receptor) analyzed with Western blotting (Δ-actin was used as loading control). **D.** Impact of Met silencing on viability of A2780 and A2780_ATO_ cells after 72 h incubation was analyzed with MTT assay. Impact of treatment on cell viability was evaluated using two-way ANOVA with Bonferroni post-test, * p < 0.05. **E.** Silencing of Met receptor with siRNA checked by Western blotting (quantification at Supplementary Figure S2C). **F.** Immunohistochemistry of A2780 and A2780_ATO_ xenograft samples stained for expression of Met and Ki67 (proliferation marker) as indicated. Representative examples are shown in three different magnifications (objective magnifications 10x, 20x and 40x). Additional immunohistochemistry examples of A2780 and A2780_ATO_ xenografts and quantification are included as Supplementary Figure S3.

As a next step, we investigated the mechanisms underlying the aggressive phenotype of A2780_ATO_ cells. First, as decreased drug influx or increased drug efflux are often observed in the course of chronic drug exposure, intracellular arsenic levels were measured by ICP-MS after 3 h of drug treatment (Supplementary Figure S1B). These experiments confirmed that the resistance of A2780_ATO_ cells is not based on enhanced drug efflux. A2780_ATO_ cells were further tested for cross-resistance against several anticancer drugs with specific modes of action and targets. The panel of tested compounds included conventional chemotherapeutic drugs like doxorubicin, cisplatin, paclitaxel, and vincristine but also diverse kinase inhibitors targeting 1) EGFR and HER2 (lapatinib), 2) VEGFR, PDGFR, and Raf (sorafenib), 3) Met (crizotinib and PHA-665752), 4) FAK (FAK inh.) or 5) SRC (SRC inh. II). Resistance factors (RF) of the respective drugs were calculated from the IC_50_ after 72 h (Figure 3A and Supplementary Table S1). A2780_ATO_ showed cross-resistance against vincristine, doxorubicin, the SRC inhibitor, and lapatinib with RFs of 3.7, 3.0, 2.1, and 1.7, respectively. There was no difference in the anticancer activity (RF~1) against the FAK inhibitor, paclitaxel, sorafenib and cisplatin. Surprisingly, A2780_ATO_ cells showed collateral sensitivity towards both crizotinib and PHA-665752 with RFs of 0.15 and 0.08, respectively (Figure 3A–3B, Supplementary Table S1 and Supplementary Figure S2A). As crizotinib is already in clinical use and exerts general superior activity as compared to PHA-665752, the majority of the subsequent experiments was performed with this Met inhibitor. In line with the collateral sensitivity, Met expression was increased in A2780_ATO_ at both mRNA and protein levels (Figure 3C). In contrast, the two other well known targets of crizotinib, namely ALK and ROS1 [23], were not expressed as indicated by very low mRNA signals detected (raw expression values for *ALK* and *ROS1* below 10; *MET* expression was 88-fold increased in A2780_ATO_ as compared to A2780) (Supplementary Figure S2B). The role of *MET* in the collateral sensitivity towards crizotinib and PHA-665752 was confirmed by siRNA transfection experiments, where specific knockdown of *MET* significantly inhibited the viability of A2780_ATO_, whereas A2780 cells were not affected (Figure 3D–3E). In accordance, analysis of immunohistochemistry staining of A2780_ATO_ xenografts revealed that Met was overexpressed also in the tumor tissues and showed a spatial positive co-expression with overexpression of the proliferation marker Ki67 (Figure 3F and Supplementary Figure S3). In contrast, A2780 xenografts were Met-negative, which indicates that proliferation of the parental line is driven by other factors than Met. Noteworthy, the highly ATO-sensitive parental line expressed by far the lowest intrinsic Met levels of all cell models investigated in our initial screening panel (Supplementary Figure S4; compare Supplementary Figure S1A). In addition, intrinsic Met expression showed a trend to inversely correlate with ATO sensitivity in the entire cell line panel (r^2^ = 0.3, p = 0.1). This relation became highly significant when removing two extremely ATO-resistant lung cancer cell lines from the regression analysis (r^2^ = 0.82, p < 0.002) suggesting that NSCLC cell lines might be characterized by additional, tissue type-specific resistance mechanisms against ATO. Summarized, these observations strongly suggest that Met receptor contributes to ATO resistance but also enhanced aggressiveness of ATO-selected A2780 cells.

### Gene expression profile of A2780_ATO_ is associated with enhanced cell proliferation and activation of biosynthesis programs

**Figure 4:**
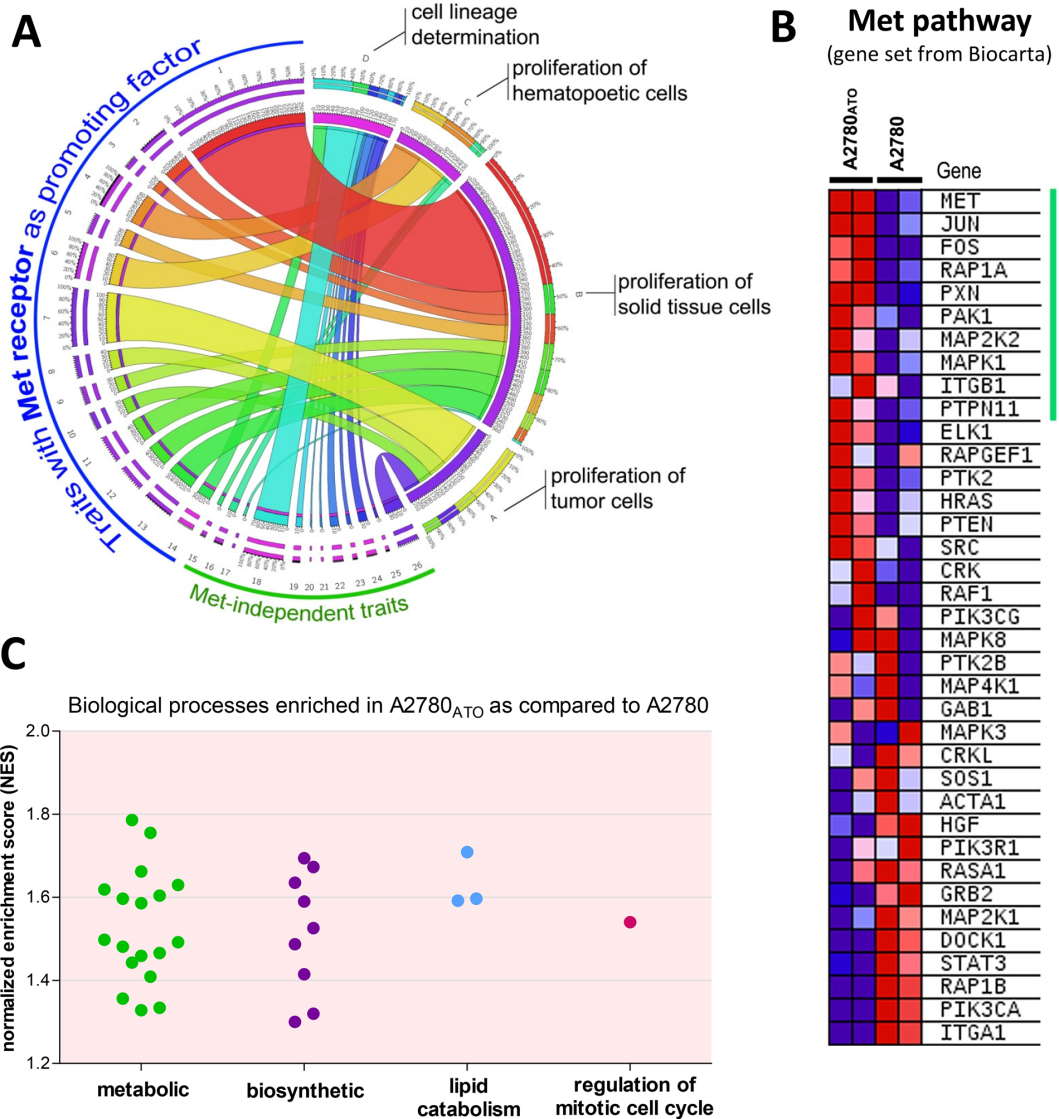
Gene set enrichment analysis of the Met pathway and the biological processes **A.** Circos plot showing the association between significantly changed traits in A2780_ATO_ vs. A2780 cells (p < 0.005; see also Supplementary Table S3) that contain Met as promoting factor and Met-independent traits predicted with IPA according to gene expression array data. The widths of the connectors represent the number of molecules predicted in the respective cancer process. The blue line indicates 14 processes that include Met as promoting factor and the green line 12 other traits without Met as a player. **B.** Heatmap from GSEA analysis of upregulated Met pathway (data set from Biocarta, National Cancer Institute, USA). Shown are respective genes of the whole data set and a subset of genes that contributes most to the upregulation of the data set (indicated with the vertical green line). **C.** Significantly enriched Gene Ontology (GO) terms (biological processes) that are upregulated in A2780_ATO_ as compared to parental cell line (see also Supplementary Table S4).

Whole genome gene expression arrays revealed that the majority of significantly differently expressed genes (631 from 806 – see data analysis in materials and methods) in the ATO-resistant subline compared to parental A2780 cells play a role in cancer (see IPA report in Supplementary Materials, Supplementary Figure S5, and Supplementary Table S2). The top 5 molecular and cellular functions influenced by the genes significantly changed in A2780_ATO_ as compared to A2780 cells were “cell death and survival”, “cellular growth”, and “proliferation” as well as “cell morphology”. *MET* appeared as one of the top upregulated genes in A2780_ATO_ and was predicted as the main promoting factor in various gene networks associated with proliferation of malignant cells, solid tissue and hematopoietic cells (Figure 4A and Supplementary Table S3). GSEA analysis of Met pathway genes showed an upregulation of the respective gene set in A2780_ATO_ as compared to A2780, with expression of *MET, JUN, FOS*, and different Met pathway transducers like *RAP1A*, *PXN, PAK1, MAP2K2, MAPK2* contributing the most to the enrichment score (Figure 4B). Regarding cell proliferation, gene sets of mitotic cell cycle regulation as well as various metabolic and biosynthetic processes of various cellular macromolecules like proteins, lipids, and carbohydrates were positively enriched in A2780_ATO_ as compared to A2780 and indicate an increased proliferative program of A2780_ATO_ (Figure 4C and Supplementary Table S4).

### ATO alters Met expression in ATO-sensitive and-resistant cells

**Figure 5:**
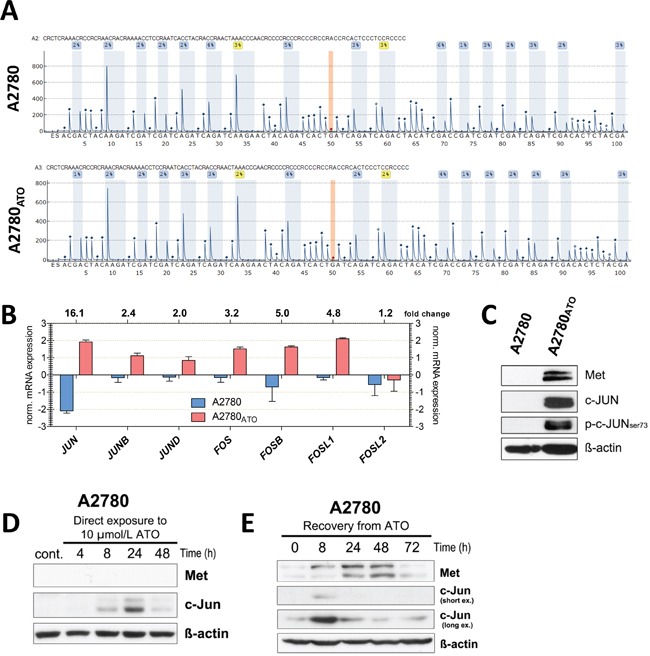
*MET* promoter methylation and targeting transcription factors **A.** Representative pyrograms for A2780 and A2780_ATO_ cell-derived DNA indicate unmethylated *MET* promoters with no difference between cell lines (all results below the limit of quantification – 5%). Peaks highlighted by blue shading: methylation levels of the CpG dinucleotides. Position 50 highlighted by orange shading: control for the completion of bisulfite treatment. **B.** Jun and Fos transcription factor gene family members were analyzed with whole genome gene expression arrays. Shown are the normalized mRNA expression values from A2780 and A2780_ATO_ cells and the change of expression. Data were evaluated with GeneSpring software (Agilent technologies). **C.** Protein expression levels of Met, c-Jun as well as phosphorylation of c-Jun at serine 73 (marker for AP-1 transactivation) investigated with Western Blot. **D.** Time-dependent impact of ATO on Met and c-Jun expression was analyzed with Western Blot during ATO exposure and **E.** in recovery phase after ATO exposure (72 h treatment with 3 μmol/L ATO, 0 h is the time of ATO removal) in A2780 cells. For all Western blot experiments, Δ-actin was used as loading control.

To investigate factors underlying Met overexpression in A2780_ATO_ cells, we analyzed the *MET* gene regulation at different levels. Array comparative genomic hybridization (array CGH) analyses revealed no change in gene copy number of A2780_ATO_ as compared to A2780 through the entire genome (Supplementary Figure S6). This finding indicates that Met expression is not altered based on a copy number gain of the *MET* gene at chromosome 7. Next, *MET* promoter methylation was analyzed by pyrosequencing. These experiments revealed that also no CpG methylation changes within the *MET* gene promoter were induced upon chronic ATO exposure (Figure 5A).

Thus, we hypothesized that ATO treatment might enhance Met transcription by activation of stress signals. Consequently, we focused on transcription factors (TFs) which might regulate *MET* gene transcription. Putative TFs binding to the *MET* promoter were predicted using the SaBiosciences database (http://www.sabiosciences.com/chipqpcrsearch.php?app=TFBS) that is based on DECipherment Of DNA Elements (DECODE). Supplementary Table S5 shows the fold change in expression of the most relevant TFs predicted to bind to the *MET* promoter in A2780_ATO_ compared to parental cells. Noteworthy, all mRNA values of the c-Jun family (*JUN, JUNB*, and *JUND*) as well as c-Fos family (*FOSB, FOSL1*, and *FOS*) were among the top upregulated *MET* promoter-binding TF-coding genes in A2780_ATO_, suggesting an important function of AP-1 in Met overexpression (Figure 5B). Among all AP-1 subunit genes, *JUN* showed the highest difference with 16.1-fold increased mRNA levels and c-Jun overexpression in Met-driven A2780_ATO_ (Figure 5C). *FOS*, *FOSB*, and *FOSL1* genes that code for dimeric partners of c-Jun for AP-1 showed 3.2-, 5.0-, and 4.8-fold enhanced mRNA expression in A2780_ATO_, respectively.

To investigate whether induction of *MET* and AP-1 family genes by ATO is also observed in other hands, we used the mRNA expression array data in the hepatocellular carcinoma cell model HepG2 treated with different concentrations of ATO for 48 h (published by Hara-Yamamura et al. in 2013 at Gene Expression Omnibus database; Accession Nr: GSE48441 [24]) and analyzed them for the respective gene expression. In line with our data on A2780 cells, also in these samples induction of *MET* expression and a concentration-dependent increase of all AP-1 family genes (except *JUND* and *FOSL2*) were found (Supplementary Figure S7A). Furthermore, we also investigated the impact of ATO on pronounced proliferation of immortalized keratinocytes (NHEK-SVTERT), and preliminary results indicate that low-level ATO treatment results in increased *JUN* and *MET* expression (Supplementary Figure S7B).

Thus, to investigate the impact of ATO on AP-1 expression in chemo-naive cells, parental A2780 were treated with ATO at several time points (Figure 5D). The highest stimulation of c-Jun expression was observed after 24 h of treatment and decreased again with prolonged incubation. Noteworthy, under direct exposure to ATO no detectable expression of Met was found over the time of 48 h. However, a rapid but transient stimulation of Met (as well as c-Jun) expression was found during recovery from ATO treatment (Figure 5E). These data indicate that Met activation might play a role in the recovery of A2780 cells surviving ATO treatment, which subsequently turns into a permanent oncogenic driver for A2780_ATO_ cells after many cycles of ATO treatment and recovery during the selection process.

### Exploitation of ATO selection-induced Met dependency as therapeutic strategy

**Figure 6:**
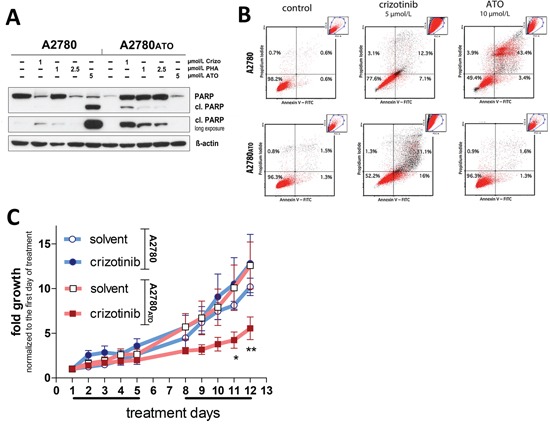
Apoptosis induction and *in vivo* antitumor activity of crizotinib against A2780 and A2780_ATO_ cells and xenografts **A.** Impact of ATO and Met inhibitors (crizotinib and PHA-665752) on PARP cleavage as a marker for apoptosis induction (Δ-actin was used as loading control). **B.** Dot plots from Annexin V / propidium iodide staining of A2780 and A2780_ATO_ measured with flow cytometry after 48 h treatment with crizotinib and ATO. **C.** Subcutaneously xenografted mice were treated with 50 mg/kg crizotinib or solvent every day, 5-times a week (indicated by the line below the x-axis), for two weeks. Treatment started after the tumors reached a measurable size. Tumor growth is presented as normalized fold growth from the first day of treatment. Statistical significance was calculated using two-way ANOVA with Bonferroni post-test (* p < 0.05; ** p < 0.01). Significance is given as comparison of the treated group with control group of the respective xenograft model.

As a next step, it was investigated, whether the ATO-induced oncogene addiction could be exploited for therapy. Figure 6A–B, as well as Supplementary Figure S8A depict that Met inhibition by crizotinib potently induced apoptosis in A2780_ATO_ cells *in vitro* indicated by caspase-mediated PARP cleavage and annexin V / PI staining. Also in SCID mice, treatment with 50 mg/kg crizotinib was well tolerated (Supplementary Figure S8B) and significantly inhibited tumor growth of A2780_ATO_ xenografts (Figure 6C), while the growth of parental A2780 tumors was not influenced by Met inhibition.

## DISCUSSION

Arsenicals represent the classical example of a double-edged sword concerning carcinogenicity and anti-cancer therapy. As one among the first active biological compounds identified, the history of arsenicals comprises many stories of curse and blessing [1, 14]. The carcinogenic potential of arsenic is well known due to exposure of millions of people to toxic levels from drinking water and food [11, 12]. Arsenicosis, as the most common phenotype of exposed people, is also the best example of arsenic-induced cell transformation. Many studies have addressed this issue and suggested different molecular mechanisms [25, 26]. Interestingly, although tumorigenic capability of chronic arsenic exposure as a single agent was not conclusively confirmed in animal models, there is strong evidence for the co-carcinogenic potential if combined with other carcinogens like UV light, N-methyl-N-nitrosourea, diepoxybutane, X-rays, methylmethane sulfonate, and tobacco [5, 11, 27]. This co-carcinogenicity of arsenic is mainly based on modulation of cellular stress responses and oxidative DNA damage [28]. In addition, the activity of arsenic is often related to direct or indirect interaction with sulfhydryl (SH) moieties of more than 200 known human proteins [11]. Thus, activation via direct interaction of arsenic with vicinal SH groups was proposed for c-SRC and other SH-rich proteins like EGFR, integrins or protein phosphatases [29]. Furthermore, direct or indirect proto-oncogene activation by arsenic exposure like KRAS in lung- and prostate epithelial cells [25, 30], c-Myc in hepatocytes [31–33] or EGFR in the lung, skin, prostate, bladder and liver tissue [16, 19, 29, 34] was described. In this study, we identified activation of oncogenic Met receptor as a new mechanism of arsenic-induced cancer aggressiveness, thus adding this protein to the list of oncogenes activated by arsenic selection. This is in line with previous observations [35], since all the oncogenic attributes found in our ATO-resistant cell line like increased cell proliferation, survival, increased tumorigenicity or a more mesenchymal phenotype are known to be activated by Met receptor signaling. Hence, Met overexpression affects many patients with various cancer types like lung, liver, breast, prostate, gastric, renal or ovarian cancers, and irrespective of treatment often correlates with poor prognosis [36, 37]. Especially in ovarian cancer, Met overexpression was identified as a prognostic marker that correlates with unfavorable prognosis associated with tumor progression [38, 39]. Various *in vitro* and *in vivo* tumor models suggest three main mechanisms of underlying constitutive and prolonged Met activation: 1) ligand-dependent mechanisms (based on ligand overexpression); 2) specific genetic lesions (gene translocations, amplifications and activating mutations); or 3) transcriptional upregulation of the Met protein [40, 41]. In our ATO-selected A2780 cell model, transcriptional upregulation is likely the mechanism of Met overexpression induced by ATO as *MET* gene amplification and promoter methylation could be excluded. In contrast, parental chemo-naive A2780 cells showed no expression of Met receptor. Thus, ligand-dependent activation was not considered to be involved. However, selective overexpression of several transcription factors known to bind and activate the *MET* gene promoter was found, pointing towards transcriptional activation. Accordingly, not only Met protein but also mRNA levels were increased in A2780_ATO_ cells. In line with these assumptions, members of the AP-1 family were among the top upregulated transcription factors in A2780_ATO_ cells. This finding is in accordance with previous studies that reveal strong evidence of AP-1 in Met transcriptional regulation [42, 43]. Within the AP-1 family, we identified c-Jun overexpression as representative part of stable AP-1 complex [44].

Generally, AP-1 is responsible for regulation of many important genes that control cell proliferation, survival, and neoplastic transformation [44]. Stimuli that activate AP-1 activity are of different nature like growth factors, pro-inflammatory cytokines or a variety of environmental stresses like short wavelength UV radiation and specific chemicals [44]. Consequently, there is plenty of evidence proving the stimulatory effect of arsenic on AP-1 activation, stability, and DNA binding [45–49]. Thus, arsenic has been shown to stimulate expression of both c-Jun and c-Fos by several molecular mechanisms. Interaction of arsenic with vicinal thiols of phosphatases allows activation of JNK signaling and finally induction of c-Jun and c-Fos [50, 51]. Additionally, arsenic induced histone H3 phosphoacetylation and H3K9 hypoacetylation specifically at the c-Fos and c-Jun chromatin sites as an instant mechanism of AP-1 activation [52, 53]. Moreover, arsenic was reported to induce malignant transformation in rat liver epithelial cells by increasing mRNA levels of *MET, HGF, JUN, MYC*, and *HRAS* next to ROS detoxifying genes (*AFP, HMOX1, SOD1, GSTP1, MT1A)* [32]. Thus, our findings on ATO-induced Met via AP-1 upregulation in the ovarian cancer model are well in agreement with previous studies.

In conclusion, we found that chronic ATO exposure of our investigated ovarian cancer cells induced stable Met receptor overexpression as oncogenic driver of tumorigenicity and aggressiveness. Thus, oncogenic Met activation by ATO exposure is an important factor that should be further investigated both in patients with ATO-resistant tumors in novel treatment regiments involving arsenic and also in patients who suffer from cancers associated with arsenic poisoning. Finally, our findings are interesting considering that Met inhibition might represent a valuable tool for treatment of Met-driven tumors induced by environmental arsenic exposure. Additionally, it needs to be further elucidated whether failure of arsenic-based therapy might be related to hyperactivation of Met-derived oncogenic signals.

## MATERIALS AND METHODS

### Chemicals

Crizotinib, sorafenib, and lapatinib were purchased from LC Laboratories (MA, USA). ATO as well as all other drugs and reagents were supplied by Sigma-Aldrich (MO, USA). ATO was dissolved in 1 mol/L NaOH at 50 mmol/L concentrated stocks. Cisplatin was dissolved in dimethylformamide (DMF) at 5 mmol/L, whereas for all other substances, dimethyl sulfoxide (DMSO) stocks at 10 – 100 mmol/L were prepared. Stock solutions were further diluted into culture media at the indicated concentrations immediately before use. The final concentrations of initial drug solvents (NaOH, DMF, and DMSO) were always less than 1%.

### Cell culture

Different human cancer cell lines were used in this study: Ovarian cancer cell line A2780 was purchased from Sigma-Aldrich (MO, USA); cervix carcinoma KB-3-1 (donated by Dr. Shen, Laboratory of Cell Biology, National Cancer Institute, Bethesda, MD, USA); colorectal carcinoma cell line HCT116 (donated by Dr. B. Vogelstein, John Hopkins University, MD, USA); melanoma cell line VM-1 was established at the Institute of Cancer Research in Vienna [54], and all the other cancer cell models were supplied by American Type Culture Collection (ATCC). HCT116 and U2OS cells were grown in McCoy's and SW1573 in DMEM culture medium. All other cell lines were cultivated in RPMI-1640. Culture media were supplemented with 10% fetal calf serum (PAA, Pasching, Austria). Cell cultures were periodically checked for contamination. Cell line authentication was performed by array CGH and/or short tandem repeat fingerprint.

### Chronic ATO exposure for resistance development

A2780_ATO_ cells were generated by periodical treatment with gradually increasing concentrations of ATO starting with 0.5 μmol/L for more than two years. ATO was administered to the cells at the day after they were passaged and cells had attached to the culture flasks. The treatment dose was increased when proliferation of cells was undisturbed upon ATO treatment for more than 2 passages.

### *In vitro* cell proliferation and cytotoxicity assays

Cell proliferation and drug activity on cell viability were observed under the inverted microscope (Nikon Eclipse Ti, Life-Cell Imaging from Visitron Systems, Germany) and measured with different colorimetric methods. For MTT assays, 2 – 4 × 10^3^ cells/well were seeded in 96-well plates and allowed to recover for 24 h. Cells were exposed to the test drugs at the indicated concentrations for 72 h. On the last day of treatment, 3-(4,5-dimethylthiazol-2-yl)-2,5-diphenyltetrazolium bromide (MTT)-based vitality assays (EZ4U; Biomedica, Vienna, Austria) were performed following the manufacturer's recommendations. For long-term treatments, cells were seeded in 24-well plates at 1.2 × 10^3^ cells/well and after 24 h recovery, they were treated for one week with the indicated drug concentrations. After drug incubation, cells were fixed with methanol for 10 min at 4°C and stained with crystal violet (1 μg/μl in phosphate-buffered saline). Quantification of cell viability was done after analyzing micrographs of the stained cells with Image J, and absorbance measurement (520 nm with TECAN absorbance reader) of dissolved crystal violet dye (dissolved in 2% sodium dodecyl sulfate). Cell viability and IC_50_ values were calculated using GraphPad Prism software (La Jolla, USA).

### Apoptosis assay

Cell death was assessed using annexin V-FITC (BD Biosciences # 556420) and propidium iodide (PI) (50 μg/ml Stock). Cell samples were double stained with 2 μl of each Annexin V-FITC/PI fluorophores and quantitatively measured using LSRFortessa flow cytometer (BD Biosciences, NJ, USA) and further analyzed using Flowing Software (University of Turku, Finland).

### Soft agar assay

Three dimensional clonogenicity was examined using two layered agar in 12-well plates. The bottom layer consisted of 0.6% agar mixed with 1xRPMI, 20% FCS. Cell suspensions (1000 and 5000) in 20% FCS-RPMI with 0.3% agar were prepared as the top layer. The number of visible spheroid colonies was assessed microscopically three weeks later by counting the number of colonies and measuring the surface according to the formula: A=a×b×π; a=length/2 and b=width/2. Images of the whole wells were captured with a Nikon D40 camera or 4x and 10x spheroid micrographs using Nikon TI300 inverted microscope.

### Promoter DNA methylation analysis by bisulfite pyrosequencing

Promoter methylation and pyrosequencing analysis was performed as previously published [55]. *MET* [NG_008996.1] promoter region was identified with the Transcriptional Regulatory Element Database (TRED). The sequences of the biotin[Btn]-labelled forward primer, the reverse primer and the sequencing primer were 5′-[Btn]GGTTGAGTTATTGGTAGGGTA-3′, 5′-CCTCCTCTCAACAAATCAACTAT-3′ and 5′-ACTCCCCATCTACTCACAAAA-3′, respectively.

### Whole genome gene expression and data analysis

Gene expression arrays were performed using 4x44K whole genome oligonucleotide-based gene expression arrays (Agilent Technologies, CA, USA) as previously published [55]. For gene expression analysis, intensity values were filtered according to sufficient (>20 raw expression values in one of the analyzed groups) and significant differences in expression (Analyzed with t-test unpaired - Benjamini-Hochberg correction, p-value cut-off:0.05). After filtering, 806 genes with significantly differing levels of expression in A2780_ATO_ compared to A2780 were further investigated with QIAGEN's Ingenuity^®^ Pathway Analysis (IPA^®^, CA, USA). Expression data for Gene Set Enrichment Analysis (GSEA) [56] were normalized in R using Robust Multi-array Average (RMA) normalization approach. Gene sets of Met pathway and biological processes analyzed were taken from GSEA MSigDB collection database (Broad Institute, MA, USA).

### RNA isolation and Real-Time PCR (RT-PCR)

Total RNA was isolated with Trizol reagent (Invitrogen, MA, USA). mRNA was reverse transcribed into cDNA and RT-PCR for relative quantification of target gene expression was performed using SYBR Green qPCR Master-Mixes (Thermo Scientific, MA, USA) on a 7500 Fast Real-time instrument (Applied Biosystems, MA, USA) following the respective instructions. Following primers were used: *MET* sense: 5′-TCCTGCAGTCAATGCCTCTC-3′ and *MET* antisense: 5′-CACATATGGTCAGCCTTGTC-3′; *JUN* sense: 5′-GGAAACGACCTTCTATGACG-3′ and *JUN* antisense: 5′-CTGCTCATCTGTCACGTTCTT-3′; *ACTB* sense: 5′-GGATGCAGAAGGAGATCACTG-3′ and *ACTB* antisense: 5′-CGATCCACACGGAGTACTTG-3′. β-actin gene *ACTB* served as reference gene.

### Gene knock-down by siRNA

Cells were transfected with Lipofectamine 2000 (Invitrogen, MA, USA) using siRNA against *MET* (Dharmacon # L-003156-00-0005) or non-targeting siRNA (Dharmacon # D-001810-10-05) following the manufacturer's recommendations. Efficacy and specificity of gene silencing was verified at the protein level by Western Blot following 72 h siRNA transfection and quantified using Image Lab software v. 5.2 (Bio-Rad laboratories, CA, USA).

### Western blot

Total protein lysates or membrane-enriched protein extracts were prepared, separated by SDS-PAGE, and transferred onto a polyvinylidene difluoride membrane for Western blotting as described previously [54]. Primary antibodies used are given in Supplementary Table S6. Secondary, horseradish peroxidase-labeled antibodies from Santa Cruz Biotechnology were used in working dilutions of 1:10 000.

### Xenograft experiments

6- to 8-week-old female CB-17 scid/scid mice were purchased from Harlan Laboratories (IN, USA). The animals were kept in a pathogen-free environment and every procedure was done in a laminar airflow cabinet. The experiments were carried out according to the regulations of the Ethics Committee for the Care and Use of Laboratory Animals at the Medical University Vienna (Vienna, Austria). For tumorigenicity, 1 × 10^6^ cells were injected subcutaneously into the right flank. Animals were controlled for distress development every day and tumor size was assessed regularly by caliper measurement. A positive tumor growth was counted if tumors were palpable and further reached volumes > 50 mm^3^ calculated by the formula: (length × width^2^)/2.

For therapeutic activity, tumor-bearing mice were randomly assigned to treatment groups and therapy was initiated only when tumors were measurable and started growing. Animals were treated orally either with 50 mg/kg crizotinib (dissolved in 5% DMSO, 10% Cremophor EL, 10% ethanol and 75% deionized water) or vehicle, 5-times a week for 2 weeks. Crizotinib was well tolerated without any change in body weight (Supplementary Figure S8B).

### Immunohistochemistry staining

Three μm paraffin-embedded tissue sections were deparaffinized and rehydrated, followed by blocking the endogenous peroxidase with 0.3% hydrogen peroxidase. After Heat Induced Antigen Retrieval (HIER) for 10 minutes in 10 mmol/L citrate buffer (pH 6.0), the tissue sections were incubated with primary rabbit mAb Met (D1C2), Cell Signaling, MA, USA, dilution 1:300, incubation time 1 h) and Ki67 mouse mAb (Dako, Glostrup, Denmark, dilution 1:100, incubation time 30 min) followed by treatment with Ultravision Labeled Horseradish peroxidase (HRP) polymer (UVLP, Dako, Glostrup, Denmark, incubation time 15 min). Antibody binding was visualized with DAB+ chromogen and counterstained with Heamatoxylin.

### Statistics

If not mentioned otherwise, data are expressed as mean ± SEM. Results were analyzed and illustrated with GraphPad Prism. Statistical analyses were performed using t-test, and two-way analysis of variance (ANOVA) with treatment, time, concentration, or cell type as independent variables. Bonferroni post-tests were conducted to examine differences between drug treatment regimens and diverse responses. P values below 0.05 were considered as statistically significant and marked with stars: * p<0.05; ** p<0.01; *** p<0.001.

## SUPPLEMENTARY FIGURES AND TABLES








