# Supplementary material for: Chronic arsenic trioxide exposure leads to enhanced aggressiveness via Met oncogene addiction in cancer cells
Source: Oncotarget. 2016 Mar 28;7(19):27379–93. doi: 10.18632/oncotarget.8415 (PMC5053657; doi:10.18632/oncotarget.8415)
Supplement: Supplementary file 1 [file oncotarget-07-27379-s001.pdf]

SUPPLEMENTARY TABLES AND FIGURES

Supplementary Table S1: Cytotoxic activity of various anticancer drugs

Drug	Conc.	A2780 (IC_{50} ± SD)	$A2780_{ATO}$ (IC_{50} ± SD)
ATO	µmol/L	1.23 ± 0.27	4.86 ± 1.38
Vincristine	nmol/L	1.13 ± 0.02	4.18 ± 0.04
Doxorubicin	µmol/L	0.012 ± 0.01	0.030 ± 0.01
SRC Inh.	µmol/L	2.19 ± 0.04	4.67 ± 0.08
Lapatinib	µmol/L	3.36 ± 0.06	5.80 ± 0.12
FAK Inh.	µmol/L	5.24 ± 0.45	5.32 ± 0.27
Paclitaxel	nmol/L	9.64 ± 0.27	9.70 ± 1.97
Sorafenib	µmol/L	3.64 ± 0.05	3.57 ± 0.16
Cisplatin	µmol/L	0.63 ± 0.10	0.58 ± 0.19
Crizotinib	µmol/L	1.17 ± 0.01	0.18 ± 0.01
PHA-665752	µmol/L	14.15 ± 0.15	0.92 ± 0.01

Supplementary Table S2: Predicted cancer types and traits as analyzed by IPA

See Supplementary File 1

Supplementary Table S3: Predicted cell growth and proliferation traits as analyzed by IPA

See Supplementary File 2

Supplementary Table S4: GO Terms (biological processes) upregulated in $A2780_{ATO}$ with a NOM $p < 0.05$

See Supplementary File 3

Supplementary Table S5: Transcription factors (TFs) predicted to bind *MET* promoter

List of top *MET* TFs upregulated in $A2780_{ATO}$ compared to A2780	
**Gene symbol**	**Fold change**
FOXF2	**73.10**
EGR4	**38.61**
JUN	**16.11**
FOXC1	**6.44**
FOSB	**4.99**
FOSL1	**4.80**
NR2F2	**3.62**
FOS	**3.17**
JUNB	**2.42**
GATA2	**2.36**
JUND	**1.96**
CUX1	**1.83**
HSF1	**1.67**
MYC	**1.64**

Supplementary Table S6: List of antibodies used for protein detection

Antigen	Antibody	Dilution	Purchased from
Met	rabbit monoclonal	1:1000	Cell Signaling # 8198
c-Jun	rabbit monoclonal	1:1000	Cell Signaling # 9165
Phospho-c-Jun (Ser73)	rabbit monoclonal	1:1000	Cell Signaling # 3270
PARP	rabbit monoclonal	1:1000	Cell Signaling # 9532
Cleaved PARP	rabbit monoclonal	1:1000	Cell Signaling # 5625
ß-actin	mouse monoclonal	1:9000	Sigma-Aldrich # A1978

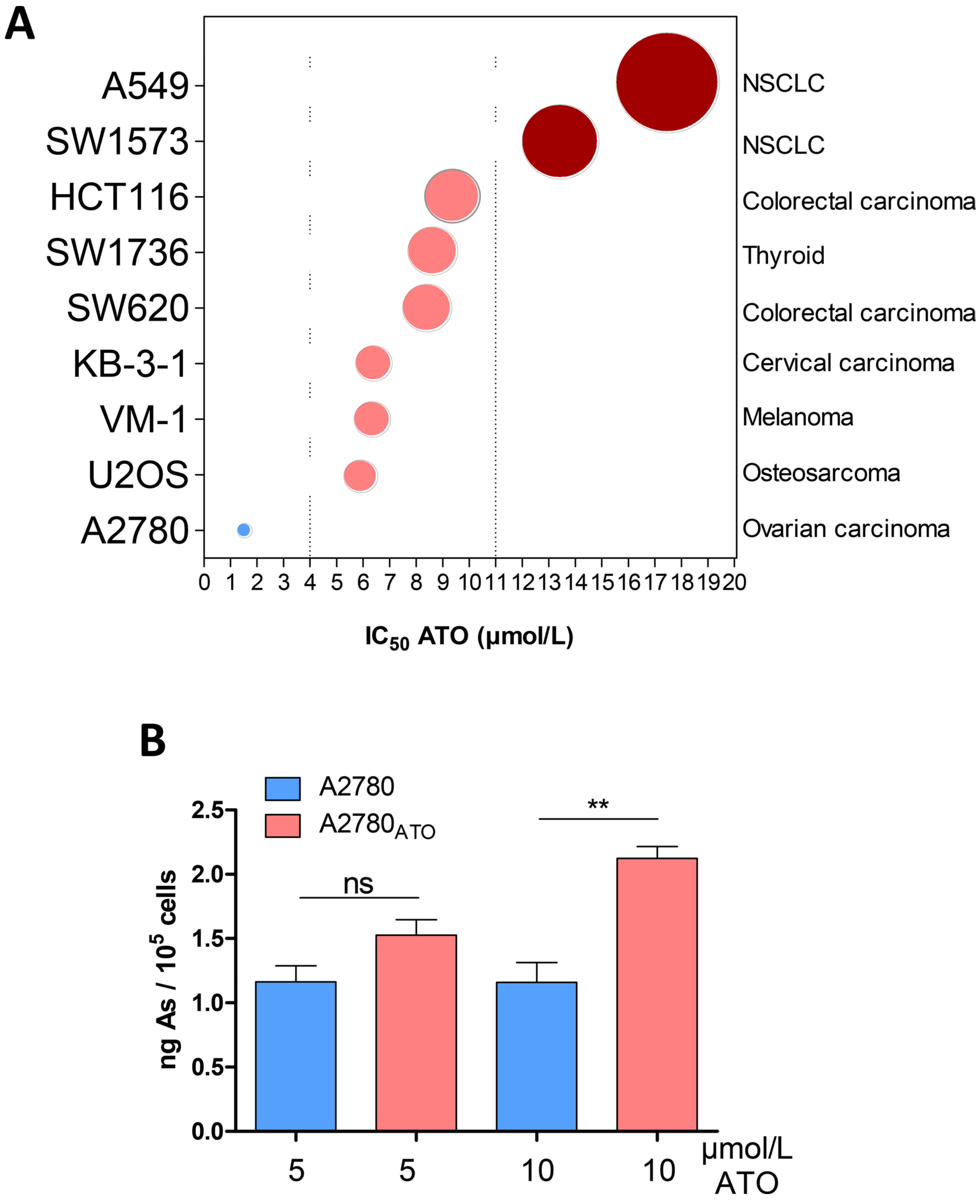

Supplementary Figure S1: Anticancer activity of ATO against different cancer cell models and intracellular arsenic (As) accumulation. A. IC_{50} values of ATO as indicated in the x-axis against the indicated cancer cell models (the size of the circles represents the ATO amount needed for the IC_{50} of the respective cell model); **B.** Intracellular As accumulation in A2780 and $A2780_{ATO}$ cells after 3 h exposure with 5 and 10 µmol/L ATO analyzed with ICP-MS, ** $p < 0.006$ analyzed with unpaired t-test.

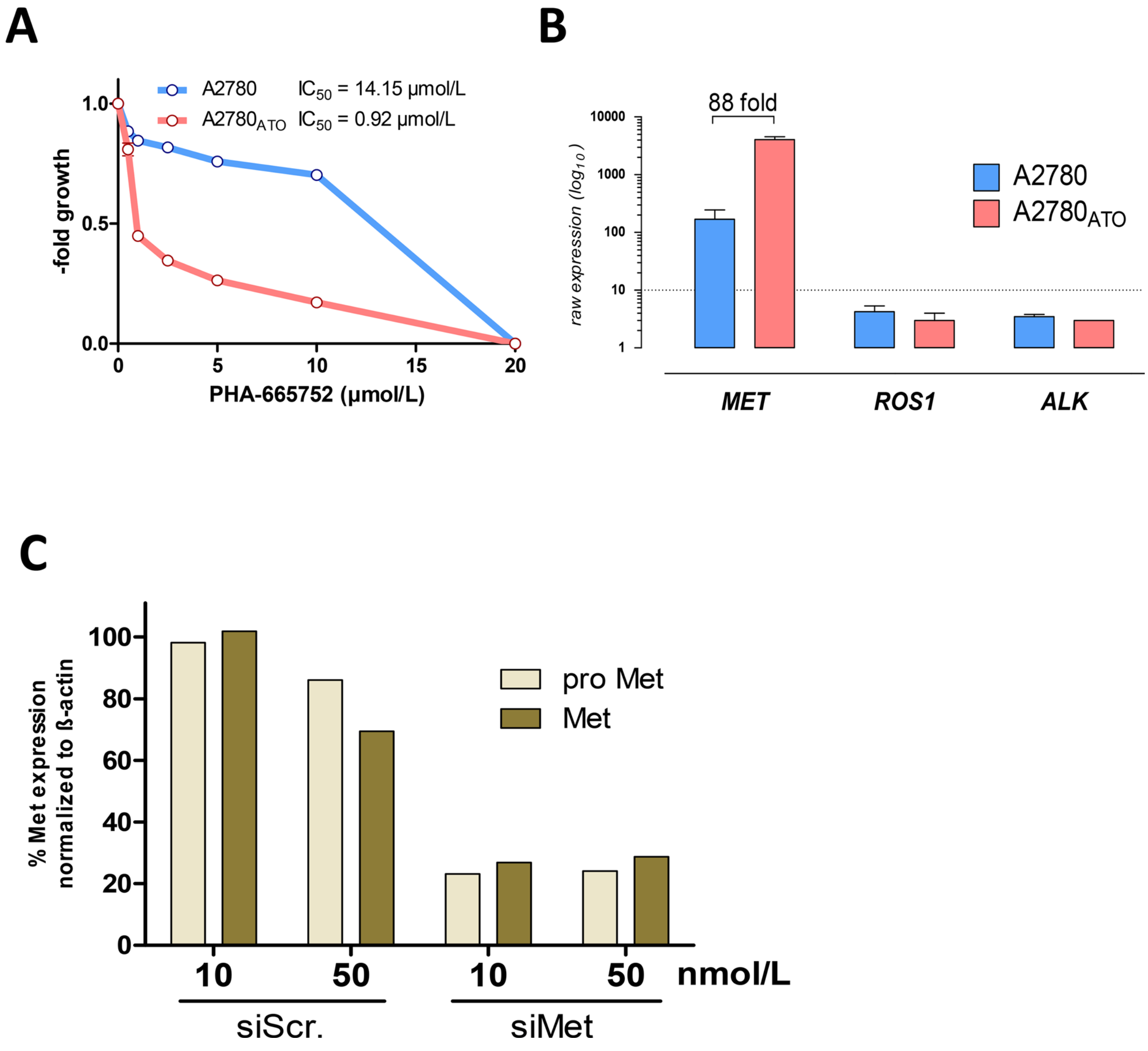

Supplementary Figure S2: Met expression and inhibition. A. Viability of A2780 and ATO-resistant subline after 72 h exposure with the indicated concentrations of PHA-665752 analyzed with MTT assay; **B.** mRNA expression (raw signal values) of crizotinib targets (Met, ROS and Alk) analyzed with whole gene expression arrays; **C.** Quantification of Western Blot bands from Met inhibition with siRNA was analyzed with ChemiDoc™ Touch. Pro Met (not-cleaved Met) and Met (mature Met) bands were quantified. ß-actin bands for normalization were analyzed with Image J.

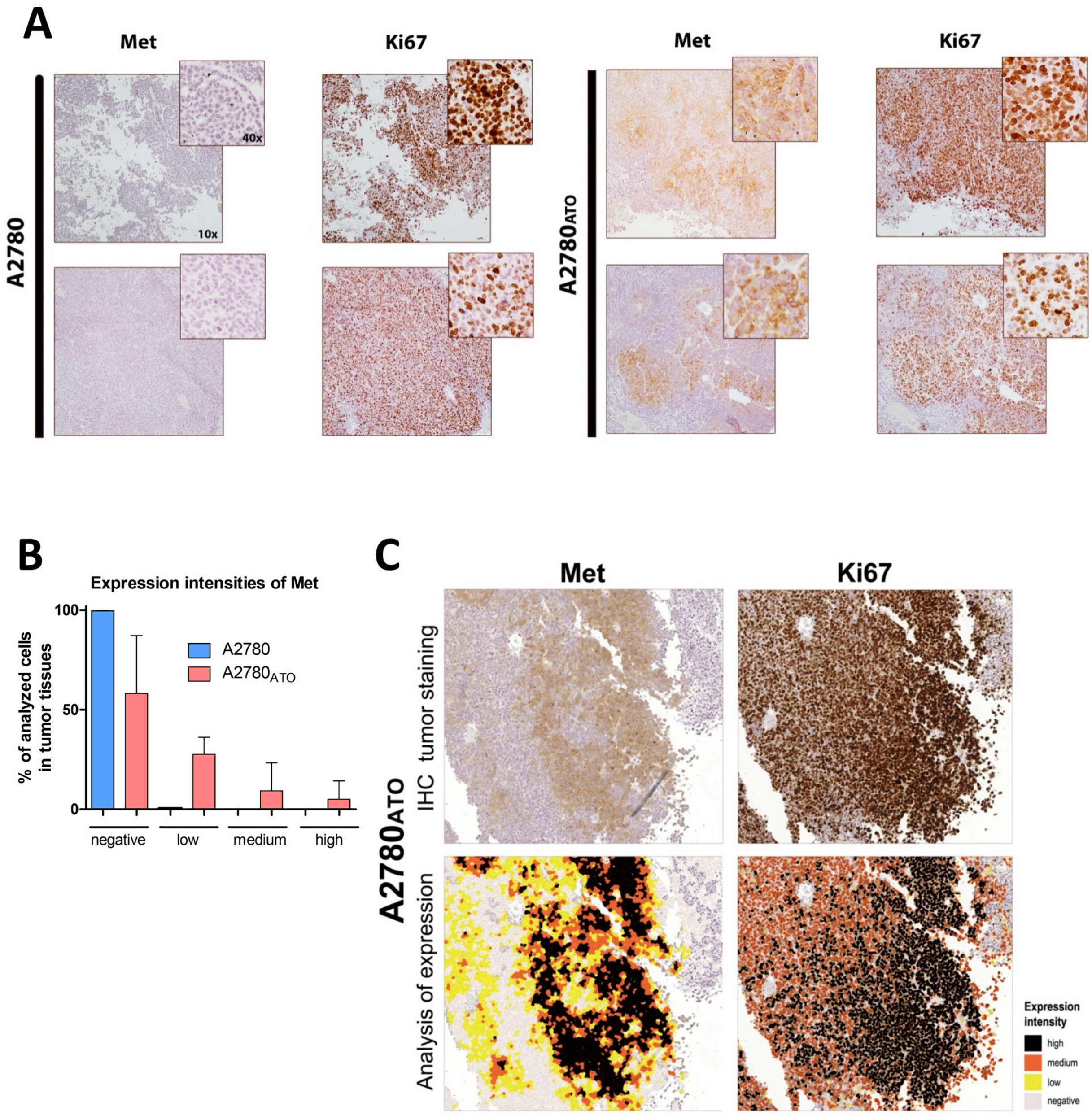

Supplementary Figure S3: Met and Ki67 expression in A2780 and A2780$_{ATO}$ xenografts. A. Immunohistochemistry staining of A2780 and A2780$_{ATO}$ xenograft samples with Met and Ki67 (proliferation marker). Representative examples are shown in two different magnifications (10x and 40x objective), **B.** Global expression analysis of Met intensities in A2780 and A2780$_{ATO}$ xenografts were identified with Definiens Tissue Studio®. **C.** A representative example of spatial expression intensities of Met and Ki67 in A2780$_{ATO}$ xenografts analyzed with Definiens Tissue Studio®.

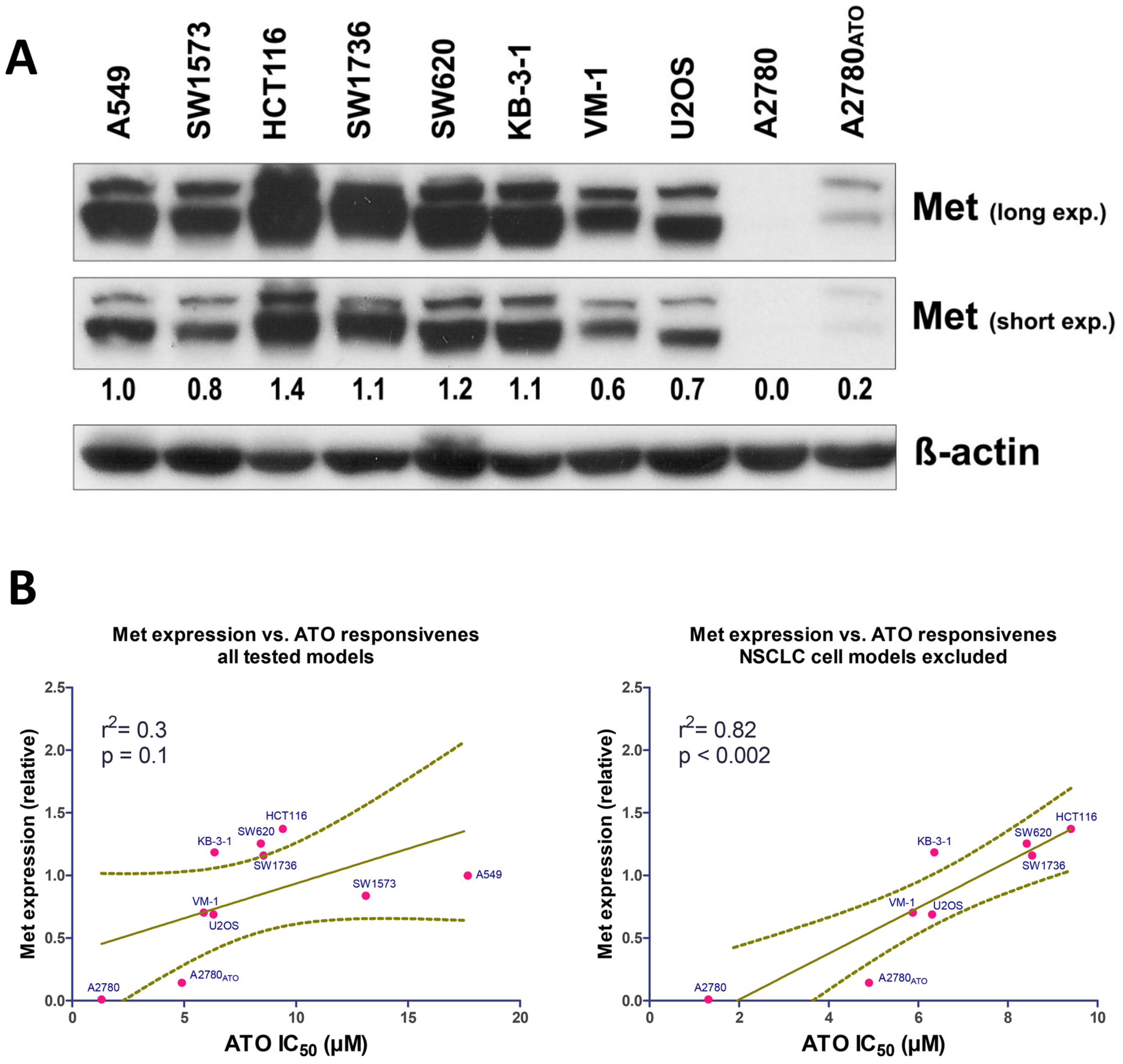

Supplementary Figure S4: Met expression versus ATO responsiveness. A. Met expression in cell lines tested for ATO responsiveness (compare Supplementary Figure S1A) analyzed with Western blotting. Relative density of Met expression bands (normalized to respective ß-actin bands; values for A549 arbitrarily set as 1) was quantified with Image J – values are shown below the respective slot. **B.** Correlation analysis (linear regression) between Met expression and ATO responsiveness. Left panel – comprises all cell models analyzed for ATO responsiveness; Right panel intrinsically ATO-hyper-resistant NSCLC cell lines (A549, SW1573) excluded.

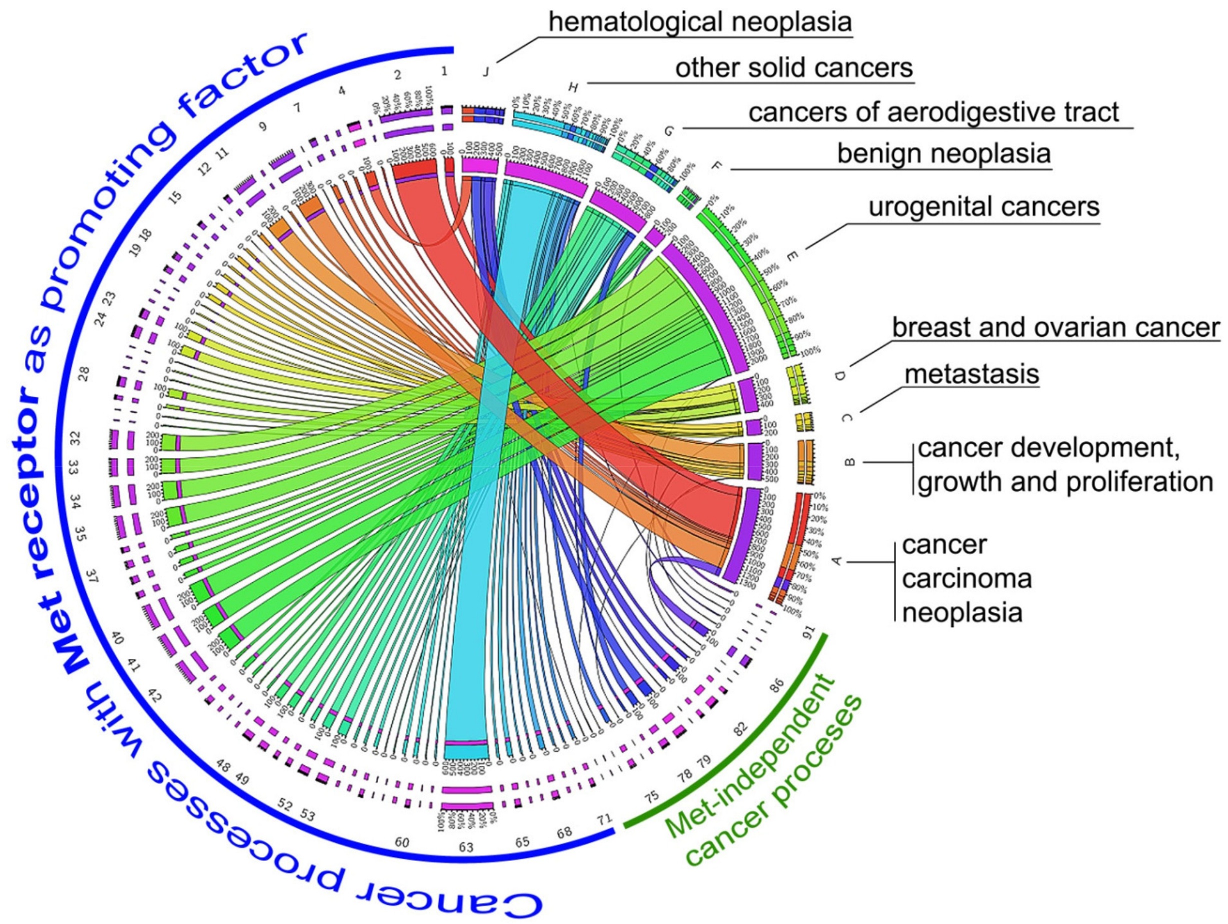

Supplementary Figure S5: Correlation of Met with different cancer types and traits. Circos plot of the association between significantly changed cancer types and traits in A2780$_{ATO}$ vs. A2780 cells ($p < 0.005$; see also Supplementary Table S2) that contain Met as promoting factor and Met independent traits predicted with IPA according to gene expression array data. The widths of the connectors represent the number of molecules predicted in the respective cancer associated process. The blue line (outer line) represents traits that include Met as promoting factor and the green line (outer line) represents other traits without Met as a player.

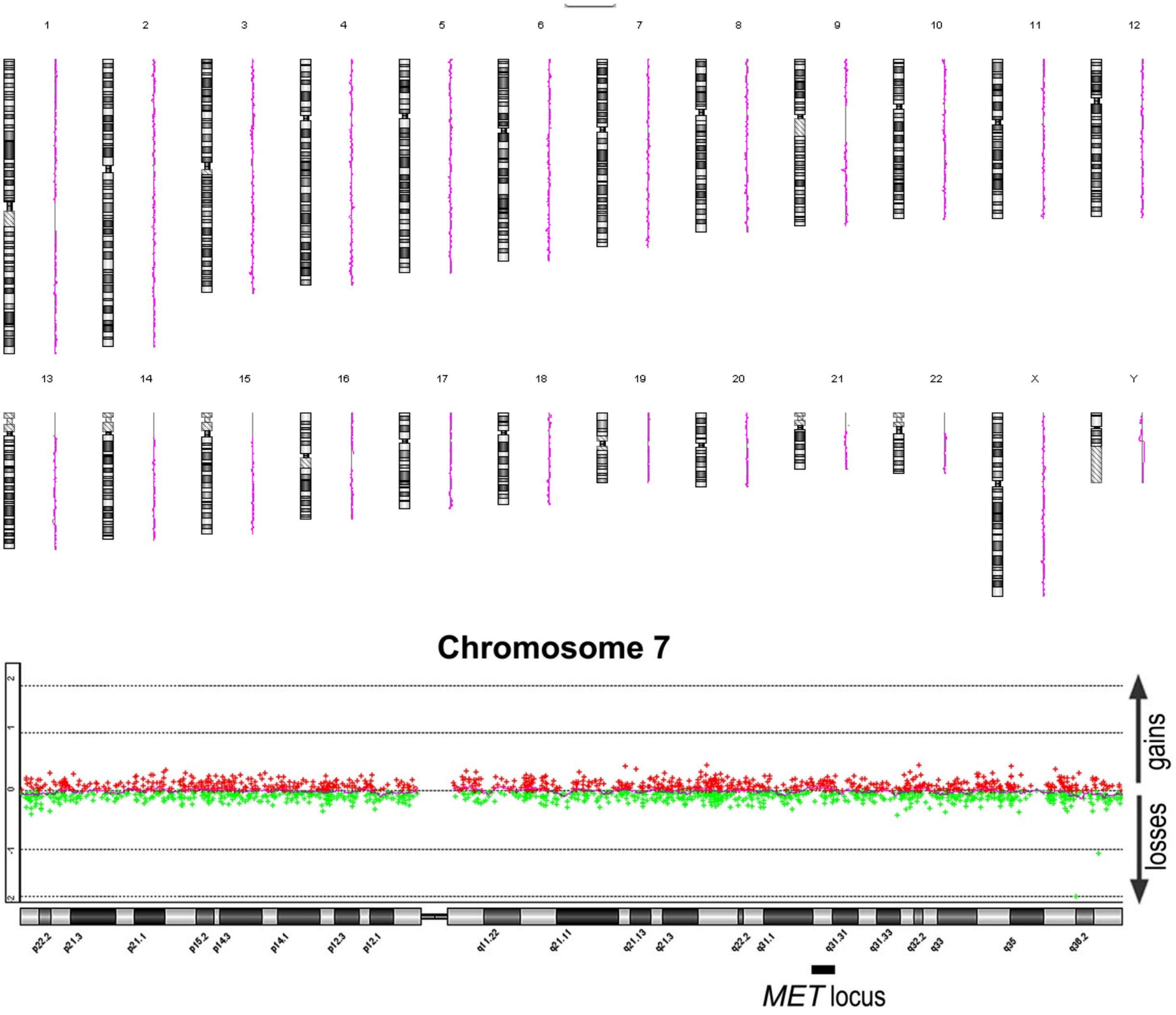

Supplementary Figure S6: array CGH analysis of A2780$_{ATO}$ as compared to A2780 cells. Chromosome profiles of whole genome array comparative genomic hybridization (indirect comparison) of A2780$_{ATO}$ and A2780 cells (upper panel) and profile of chromosome 7 including the *MET* locus as indicated (lower panel).

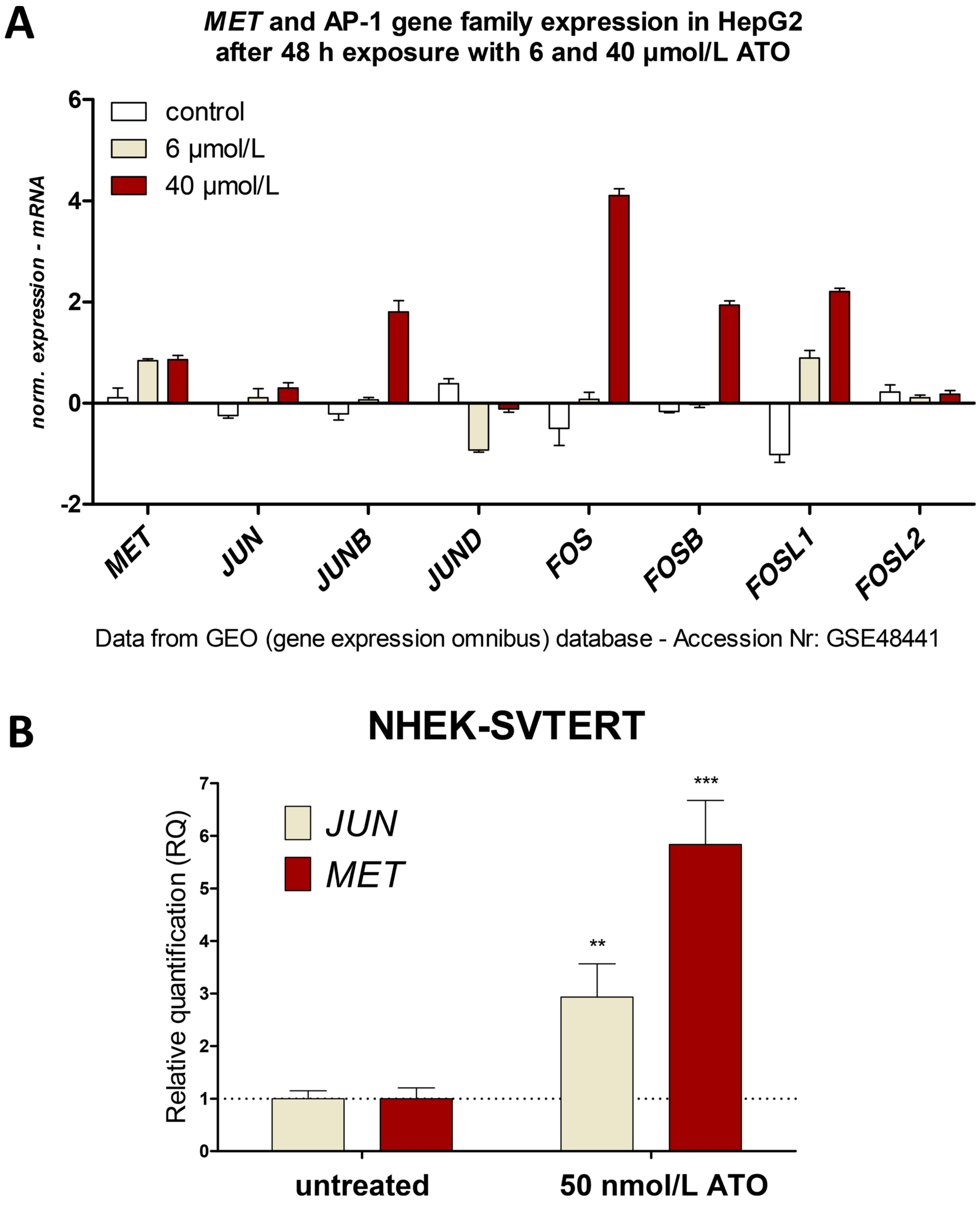

Supplementary Figure S7: *MET* and AP-1 gene family expression in HepG2 or immortalized keratinocytes after ATO treatment. A. Expression of *MET* and AP-1 gene family members analyzed with gene expression arrays (data taken from GEO database: GSE48441). Shown are the normalized mRNA expression values from untreated HepG2 cells (hepatocellular carcinoma cell line) and HepG2 treated with 6 and 40 µmol/L ATO for 48 h. Data were analyzed with GeneSpring software (Agilent technologies). **B.** *MET* and *JUN* mRNA expression in NHEK-SVTERT (immortalized keratinocytes) treated with 50 nmol/L ATO for two months and analyzed with RT-PCR (normalized to *ACTB* gene expression, *JUN* and *MET* mRNAs compared to respective untreated control, t-test, ** $p < 0.005$, *** $p < 0.001$).

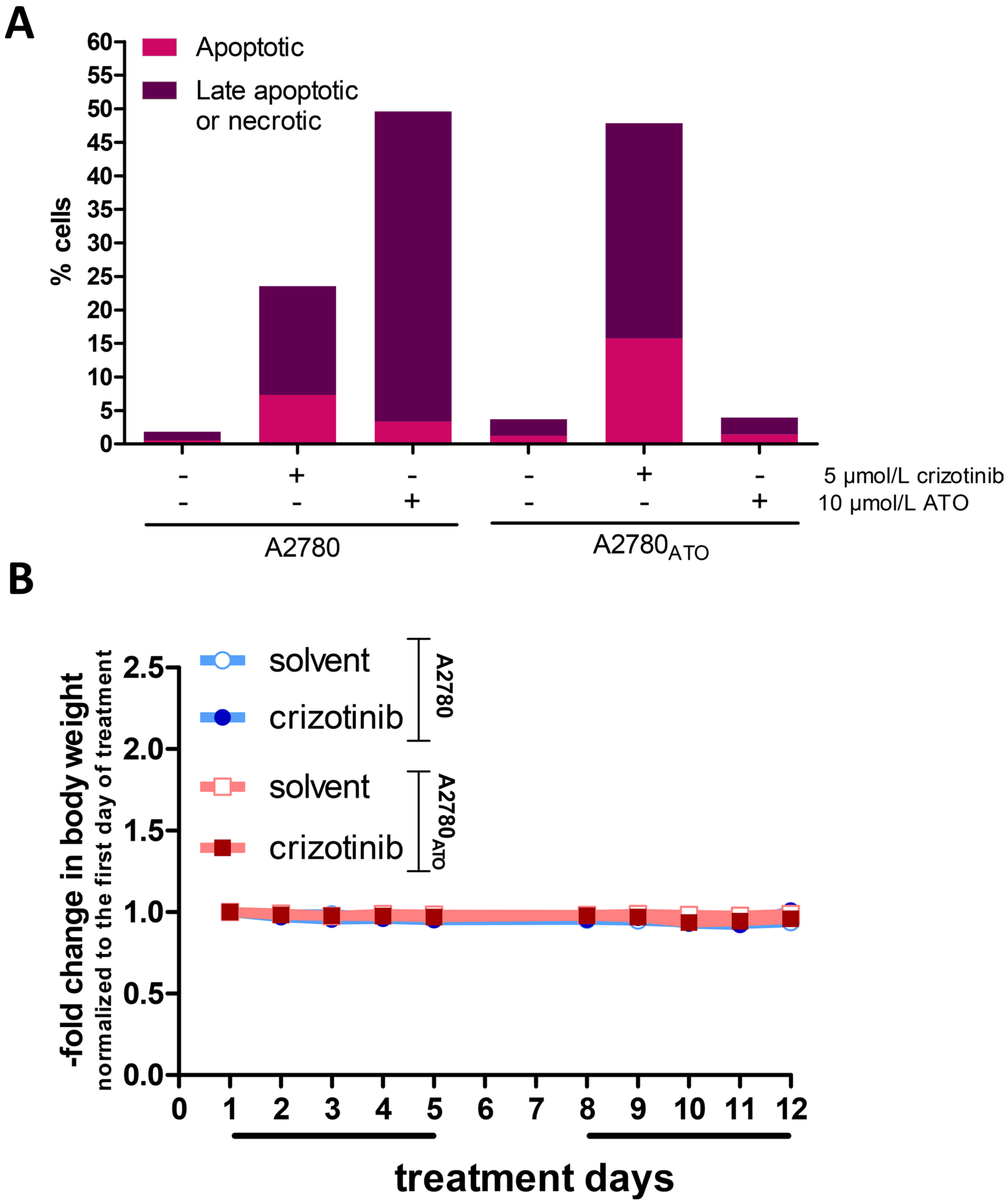

Supplementary Figure S8: Apoptosis induction and body weight of mice during treatment. **A.** Diagram of cell death (apoptosis, late apoptosis or necrosis) induction in A2780 and $A2780_{ATO}$ after treatment with 5 µmol/L crizotinib and 10 µmol/L ATO analyzed with annexin V/PI staining; **B.** Body weight of xenografted mice as an indicator of therapy side effects during treatment with 50 mg/kg crizotinib or solvent.
