# Supplementary material for: Chronic arsenic trioxide exposure leads to enhanced aggressiveness via Met oncogene addiction in cancer cells
Source: Oncotarget. 2016 Mar 28;7(19):27379–93. doi: 10.18632/oncotarget.8415 (PMC5053657; doi:10.18632/oncotarget.8415)
Supplement: Supplementary file 2 [file oncotarget-07-27379-s002.doc]

| **Table S2. Predicted cancer types and traits as analyzed by IPA. © 2000-2015 QIAGEN. All rights reserved.** | | | | | |
| --- | --- | --- | --- | --- | --- |
| **Category** | **Lalbel im circos** | **Function** | **ID** | **p-value** | **# Molecules** |
| **Cancer** | 1 | **advanced malignant tumor** | C | 7.87E-07 | 76 |
|  | 2 | **metastasis** | C | 3.39E-06 | 68 |
|  | 3 | **benign connective or soft tissue neoplasm** | F | 3.43E-06 | 37 |
|  | 4 | **hematological neoplasia** | A | 6.00E-06 | 146 |
|  | 5 | **benign neoplasia** | F | 8.86E-06 | 71 |
|  | 6 | **benign neoplasm of female genital organ** | F | 1.33E-05 | 38 |
|  | 7 | **male genital neoplasm** | E | 2.65E-05 | 82 |
|  | 8 | **neoplasia** | A | 2.68E-05 | 41 |
|  | 9 | **neoplasia** | A | 2.69E-05 | 31 |
|  | 10 | **smooth muscle tumor** | H | 3.23E-05 | 32 |
|  | 11 | **urogenital cancer** | E | 3.61E-05 | 292 |
|  | 12 | **malignant neoplasm of male genital organ** | E | 4.80E-05 | 78 |
|  | 13 | **tumorigenesis** | B | 5.67E-05 | 29 |
|  | 14 | **leiomyomatosis** | F | 5.70E-05 | 30 |
|  | 15 | **neoplasia** | A | 6.93E-05 | 76 |
|  | 16 | **genital tumor** | E | 1.00E-04 | 265 |
|  | 17 | **upper gastrointestinal tract tumor** | G | 1.06E-04 | 129 |
|  | 18 | **muscle tumor** | H | 1.31E-04 | 35 |
|  | 19 | **malignant solid tumor** | H | 1.47E-04 | 24 |
|  | 20 | **solid tumor** | H | 1.47E-04 | 24 |
|  | 21 | **carcinoma** | A | 1.53E-04 | 23 |
|  | 22 | **tumorigenesis** | B | 1.58E-04 | 255 |
|  | 23 | **upper gastrointestinal tract cancer** | G | 1.74E-04 | 123 |
|  | 24 | **genital tract cancer** | E | 1.94E-04 | 252 |
|  | 25 | **prostate cancer** | E | 1.99E-04 | 72 |
|  | 26 | **malignant neoplasm of aerodigestive tract** | G | 2.43E-04 | 54 |
|  | 27 | **gastro-esophageal carcinoma** | G | 2.63E-04 | 105 |
|  | 28 | **benign ovarian tumor** | F | 3.22E-04 | 16 |
|  | 29 | **connective or soft tissue tumor** | H | 3.57E-04 | 54 |
|  | 30 | **papillary carcinoma** | H | 3.90E-04 | 25 |
|  | 31 | **breast or ovarian cancer** | D | 4.25E-04 | 134 |
|  | 32 | **upper aerodigestive tract carcinoma** | G | 4.56E-04 | 48 |
|  | 33 | **growth** | B | 5.04E-04 | 38 |
|  | 34 | **gastroesophageal cancer** | G | 5.04E-04 | 110 |
|  | 35 | **estrogen receptor-negative breast cancer** | D | 5.18E-04 | 17 |
|  | 36 | **serous ovarian adenocarcinoma** | D | 5.58E-04 | 12 |
|  | 37 | **growth** | B | 6.20E-04 | 62 |
|  | 38 | **upper airway cancer** | G | 6.74E-04 | 13 |
|  | 39 | **proliferation** | B | 7.12E-04 | 40 |
|  | 40 | **serous ovarian carcinoma** | D | 7.21E-04 | 15 |
|  | 41 | **esophageal carcinoma** | G | 7.58E-04 | 34 |
|  | 42 | **pelvic tumor** | E | 7.97E-04 | 275 |
|  | 43 | **respiratory system tumor** | H | 8.28E-04 | 78 |
|  | 44 | **papillary adenocarcinoma** | H | 9.47E-04 | 23 |
|  | 45 | **proliferation** | B | 9.55E-04 | 32 |
|  | 46 | **HER2 negative hormone receptor negative breast cancer** | D | 9.65E-04 | 16 |
|  | 47 | **neoplasia** | A | 9.98E-04 | 7 |
|  | 48 | **tumorigenesis** | B | 1.32E-03 | 58 |
|  | 49 | **pelvic cancer** | E | 1.34E-03 | 272 |
|  | 50 | **nasopharyngeal carcinoma** | G | 1.36E-03 | 7 |
|  | 51 | **breast cancer** | D | 1.84E-03 | 106 |
|  | 52 | **gonadal tumor** | E | 1.95E-03 | 58 |
|  | 53 | **metastasis** | C | 2.07E-03 | 17 |
|  | 54 | **esophageal cancer** | G | 2.16E-03 | 36 |
|  | 55 | **cancer** | A | 2.17E-03 | 131 |
|  | 56 | **female genital neoplasm** | E | 2.41E-03 | 226 |
|  | 57 | **mammary tumor** | D | 2.50E-03 | 113 |
|  | 58 | **epithelial ovarian cancer** | D | 2.58E-03 | 13 |
|  | 59 | **metastasis** | C | 3.17E-03 | 21 |
|  | 60 | **metastatic solid tumor** | C | 3.77E-03 | 24 |
|  | 61 | **squamous-cell carcinoma** | H | 3.91E-03 | 52 |
|  | 62 | **female genital tract cancer** | E | 4.24E-03 | 213 |
|  | 63 | **ovarian cancer** | D | 4.30E-03 | 49 |
|  | 64 | **neoplasia** | A | 4.51E-03 | 308 |
|  | 65 | **differentiated thyroid cancer** | H | 4.55E-03 | 12 |
|  | 66 | **clear-cell adenocarcinoma** | H | 4.68E-03 | 44 |
|  | 67 | **cancer** | A | 4.72E-03 | 628 |
|  | 68 | **myeloid neoplasm** | H | 4.82E-03 | 69 |
|  | 69 | **development** | B | 5.03E-03 | 2 |
|  | 70 | **malignant solid tumor** | H | 5.21E-03 | 622 |
|  | 71 | **renal cancer** | G | 5.36E-03 | 60 |
|  | 72 | hematologic cancer | J | 3.06E-05 | 138 |
|  | 73 | gastroesophageal adenocarcinoma | G | 3.42E-04 | 100 |
|  | 74 | neoplasia | A | 3.43E-04 | 126 |
|  | 75 | lymphoid cancer | J | 1.39E-03 | 118 |
|  | 76 | uterine leiomyoma | F | 1.64E-03 | 24 |
|  | 77 | skin carcinoma | H | 1.81E-03 | 13 |
|  | 78 | transformation | B | 2.79E-03 | 4 |
|  | 79 | lymphoreticular neoplasm | J | 2.81E-03 | 51 |
|  | 80 | secondary neoplasm of urinary system | E | 3.24E-03 | 5 |
|  | 81 | apoptosis | J | 3.42E-03 | 3 |
|  | 82 | tumorigenesis | B | 3.42E-03 | 3 |
|  | 83 | basal-cell carcinoma | H | 3.80E-03 | 9 |
|  | 84 | metastasis | C | 3.89E-03 | 6 |
|  | 85 | esophageal adenocarcinoma | G | 4.03E-03 | 29 |
|  | 86 | lymphocytic cancer | J | 4.18E-03 | 50 |
|  | 87 | bone marrow neoplasm | H | 4.44E-03 | 69 |
|  | 88 | interphase | J | 4.60E-03 | 5 |
|  | 89 | metastasis | C | 4.97E-03 | 3 |
|  | 90 | bladder cancer | E | 5.03E-03 | 2 |
|  | 91 | cell cycle progression | B | 5.03E-03 | 2 |
| **Process in Circos plot: A - cancer, carcinoma, neoplasia; B - cancer development; growth and proliferation; C - metastasis; D - breast and ovarian cancer; E - urogenital cancers; F - benign neoplasia; G - cancers of aerodigestive tract; H - other solid cancers; J - hematological neoplasia** | | | | | |
