# Supplementary material for: Chronic arsenic trioxide exposure leads to enhanced aggressiveness via Met oncogene addiction in cancer cells
Source: Oncotarget. 2016 Mar 28;7(19):27379–93. doi: 10.18632/oncotarget.8415 (PMC5053657; doi:10.18632/oncotarget.8415)
Supplement: Supplementary file 3 [file oncotarget-07-27379-s003.doc]

| **Table S3. Predicted cell growth and proliferation traits as analyzed by IPA ^© 2000-2015 QIAGEN. All rights reserved.^** | | | | | | |  |
| --- | --- | --- | --- | --- | --- | --- | --- |
| **Category** | **Nr:** | **ID:** | **Function Annotation** | **p-value** | **Molecules** | **# Molecules** | |
| **Cellular Growth and Proliferation** | 1 | B | **proliferation of cells** | 2.21E-06 | AATK, ABCG1, ADCYAP1, AK4, AKAP12, AKR1C3, AMFR, AMOT, ANK3, ANXA1, ARHGDIB, ASCL2, AZIN2, B4GALT1, BAMBI, BLM, BMPER, CACFD1, CAPRIN1, CASP1, CASP7, CASZ1, CAV1, CAV2, CBFA2T3, CBX2, CCNG2, CCPG1, CD24, CD276, CD70, CD83, CDCA7L, CDH2, CDH23, CEP131, CES2, CITED2, CLDN11, CLMN, CNOT6L, COMP, COPS8, CRIP1, CSF2RA, CTSC, CXCL16, CXCR4, CYLD, CYP1B1, DACT3, DAPK2, DCLK1, DCN, DDR2, DLK1, DLX6, DUSP12, DVL1, E2F2, EGR1, EGR2, EMILIN2, EOMES, EPOR, ERBB4, ERCC1, ERRFI1, ETV1, F2R, FABP5, FANCL, FBLN1, FBN2, FERMT1, FGF20, FGFR3, FGL1, FOXC1, FTL, FZD3, FZD5, GAD1, GCLC, GDNF, GDPD5, GMEB1, GPER1, GPM6A, GPR158, GREM1, GSN, GYLTL1B, HDAC2, HEY1, HIC1, HIF1A, HK2, HOXB5, HSD11B2, HSPA1A/HSPA1B, ICOSLG/LOC102723996, IDO1, IER3, IFIT3, IGFBP6, IKZF2, IL27RA, INPP5D, IRF1, ITGA1, ITGA4, ITGB5, JAG1, KDM6A, KIF1A, KIF3C, KIFAP3, KLF10, KLF3, KMT2C, LAMA1, LZTS1, MAB21L1, MAGED1, MAP2K6, MARCKS, MARCKSL1, MEF2C, MEIS1, **MET**, MIR17HG, MLLT3, MMP1, NDRG1, NEDD4L, NFAT5, NPR3, NPTX2, NR6A1, NREP, NRK, NRXN1, NTN1, NTS, NUAK1, OSR2, OSTF1, OXTR, P3H2, PALLD, PAPSS2, PAX6, PBX1, PBX3, PDGFA, PDGFRB, PDIA5, PDLIM4, PDP1, PHOX2B, PICALM, PIK3CA, PIM1, PITPNA, PITX2, PLAG1, PLAT, PLAUR, PLCE1, PLK5, PML, POC1B, POU3F2, POU4F1, PPAT, PPP1R1C, PPP1R3A, PPP3CA, PRAME, PRKD1, PRKD3, PRRX1, PTGER2, PTGS2, PTH1R, PTPN3, PTPRU, PTX3, RAD21, RANBP17, RBFOX2, RBL1, RBP1, RCAN1, RECK, RERG, RET, RGCC, RHOH, RIMS3, ROCK1, SATB1, SCN1B, SEC14L2, SETBP1, SHC4, SLC1A3, SLC2A1, SLC7A11, SMARCA1, SNCA, SOCS3, SOD2, SOX4, SOX9, SRGAP2, STAT1, STAT2, STEAP3, STIM1, STK17B, SUFU, SYNM, TAC1, TCF7, TERF1, TESC, TIMP3, TJP2, TLE4, TLR3, TMEM2, TMSB10/TMSB4X, TNFAIP8, TNFRSF14, TRPC4, TXN, TYRP1, UBE2N, UBE4B, UPP1, USP18, USP3, UTF1, UTRN, VAPA, VCAN, VDR, VGF, WNT3, WNT3A, ZBTB5, ZEB1, ZFP36L1, ZNF521 | 256 | |
|  | 2 | B | **proliferation of connective tissue cells** | 2.47E-05 | AKAP12, CAV1, CBX2, CCNG2, CITED2, DCN, DDR2, ERBB4, ERRFI1, F2R, FBLN1, FERMT1, FGFR3, FOXC1, GPER1, GREM1, HIF1A, HSPA1A/HSPA1B, IDO1, IGFBP6, ITGA1, KLF10, LZTS1, MAP2K6, **MET**, NDRG1, NPR3, OSR2, PBX1, PDGFA, PDGFRB, PIK3CA, PITPNA, PLAT, PLAUR, PLCE1, PML, PPAT, PRKD1, PTGER2, PTGS2, PTH1R, RBL1, RECK, RET, SLC7A11, SOCS3, SOD2, STAT1, TAC1, TNFAIP8, TXN, VCAN, VDR, WNT3A | 55 | |
|  | 3 | B | **proliferation of epithelial cell lines** | 1.39E-04 | CD24, EGR1, ERBB4, FBN2, FGFR3, HSPA1A/HSPA1B, ITGA1, LZTS1, MAGED1, **MET**, NTN1, NTS, OXTR, PBX1, PIK3CA, PLAUR, PTGER2, PTGS2, RET, SEC14L2, SOCS3, TAC1, TRPC4, ZBTB5 | 24 | |
|  | 4 | C | **proliferation of immune cells** | 2.04E-04 | ABCG1, ADCYAP1, ANXA1, ARHGDIB, BLM, CAV1, CBX2, CD24, CD276, CD70, CD83, CSF2RA, CXCL16, CXCR4, CYLD, DCN, E2F2, EGR1, EGR2, EOMES, ERCC1, FTL, GAD1, HIF1A, HSPA1A/HSPA1B, ICOSLG/LOC102723996, IDO1, IKZF2, IL27RA, INPP5D, IRF1, JAG1, KLF3, MAP2K6, MEF2C, MEIS1, **MET**, MIR17HG, MLLT3, NFAT5, PBX1, PIK3CA, PIM1, PML, PPAT, PPP3CA, PRKD1, PTGS2, RCAN1, RHOH, SATB1, SETBP1, SOCS3, STAT1, STK17B, TAC1, TCF7, TLR3, TNFRSF14, TXN, TYRP1, UBE2N, VDR, WNT3A, ZFP36L1 | 65 | |
|  | 5 | B | **proliferation of fibroblast cell lines** | 3.07E-04 | AKAP12, BLM, CAPRIN1, CAV1, CES2, CNOT6L, CSF2RA, E2F2, EGR1, EGR2, ERBB4, ERRFI1, ETV1, F2R, FGFR3, GDNF, HSPA1A/HSPA1B, IER3, INPP5D, IRF1, JAG1, LZTS1, MAP2K6, **MET**, NREP, PBX1, PDGFA, PIK3CA, PLAG1, PLAUR, PLCE1, PML, RBL1, RET, SOX9, STAT1, VCAN, WNT3A | 38 | |
|  | 6 | C | **proliferation of blood cells** | 4.37E-04 | ABCG1, ADCYAP1, ANXA1, ARHGDIB, BLM, CAV1, CBX2, CD24, CD276, CD70, CD83, CSF2RA, CXCL16, CXCR4, CYLD, DCN, E2F2, EGR1, EGR2, EOMES, EPOR, ERCC1, FTL, GAD1, HIF1A, HSPA1A/HSPA1B, ICOSLG/LOC102723996, IDO1, IKZF2, IL27RA, INPP5D, IRF1, JAG1, KLF3, MAP2K6, MEF2C, MEIS1, **MET**, MIR17HG, MLLT3, NFAT5, PBX1, PIK3CA, PIM1, PML, PPAT, PPP3CA, PRKD1, PTGS2, RCAN1, RHOH, SATB1, SETBP1, SOCS3, STAT1, STK17B, TAC1, TCF7, TIMP3, TLR3, TNFRSF14, TXN, TYRP1, UBE2N, VDR, WNT3A, ZFP36L1 | 67 | |
|  | 7 | A | **proliferation of tumor cell lines** | 5.24E-04 | ADCYAP1, AKAP12, AKR1C3, AMOT, ANXA1, AZIN2, CASP1, CASP7, CAV1, CCNG2, CD24, CES2, COMP, COPS8, CXCR4, CYP1B1, DAPK2, DCN, DLK1, DVL1, EGR1, EGR2, EMILIN2, EPOR, ERBB4, ERCC1, ERRFI1, ETV1, FABP5, FGFR3, FOXC1, FTL, GDNF, GPER1, GYLTL1B, HDAC2, HIC1, HIF1A, HK2, HSD11B2, IDO1, IER3, INPP5D, IRF1, ITGA1, ITGB5, JAG1, KIF1A, KLF10, LZTS1, MAGED1, MAP2K6, **MET**, MIR17HG, MLLT3, MMP1, NDRG1, NTS, OXTR, PBX3, PDGFA, PDGFRB, PDLIM4, PDP1, PIK3CA, PIM1, PLAT, PLAUR, PML, POU4F1, PRAME, PRKD1, PRKD3, PTGER2, PTGS2, PTPRU, RAD21, RANBP17, RBL1, RET, SATB1, SLC2A1, SLC7A11, SOCS3, SOD2, SOX4, SOX9, STAT1, STAT2, STEAP3, STIM1, SUFU, SYNM, TAC1, TERF1, TIMP3, TLE4, TMEM2, TMSB10/TMSB4X, TNFAIP8, TXN, USP18, UTRN, VCAN, VDR, WNT3A, ZEB1 | 107 | |
|  | 8 | A | **proliferation of tumor cells** | 7.12E-04 | ANXA1, BAMBI, CASP1, CAV1, CD70, CSF2RA, CXCR4, DCN, DLK1, EGR1, F2R, FGFR3, GDNF, GREM1, HDAC2, HIF1A, HSPA1A/HSPA1B, ITGA1, JAG1, LZTS1, **MET,** MIR17HG, NDRG1, NTS, PIM1, PLAT, PLAUR, PLCE1, POU3F2, PTGS2, RCAN1, RET, SEC14L2, SOCS3, STAT1, TAC1, TIMP3, VCAN, WNT3, WNT3A | 40 | |
|  | 9 | B | **proliferation of fibroblasts** | 7.50E-04 | CAV1, CBX2, CITED2, DCN, DDR2, FOXC1, GPER1, HIF1A, ITGA1, LZTS1, MAP2K6, **MET,** NDRG1, NPR3, PDGFA, PIK3CA, PITPNA, PLAT, PLCE1, PML, PPAT, PTGER2, RBL1, SLC7A11, SOCS3, SOD2, STAT1, TAC1, TNFAIP8, TXN, VCAN, WNT3A | 32 | |
|  | 10 | A | **proliferation of cancer cells** | 9.55E-04 | BAMBI, CASP1, CAV1, CD70, CSF2RA, DCN, DLK1, EGR1, F2R, FGFR3, GDNF, GREM1, HDAC2, HIF1A, ITGA1, JAG1, LZTS1, **MET,** MIR17HG, NDRG1, PIM1, PLAT, PLAUR, PLCE1, POU3F2, PTGS2, RCAN1, RET, SEC14L2, SOCS3, STAT1, WNT3A | 32 | |
|  | 11 | B | **proliferation of neuronal cells** | 1.69E-03 | AATK, ADCYAP1, ANK3, CAV1, CDH2, DCLK1, DVL1, EGR1, ERBB4, FGFR3, FZD3, GDNF, GPM6A, HIF1A, ITGA1, ITGA4, JAG1, KIF3C, LAMA1, MARCKS, **MET,** MIR17HG, NEDD4L, NRXN1, NTN1, PALLD, PAX6, PDGFRB, PHOX2B, PIK3CA, PLAT, POU4F1, RBL1, RET, RIMS3, ROCK1, SCN1B, SOCS3, SRGAP2, STIM1, TXN, UBE4B, VAPA, VCAN, VGF, WNT3, WNT3A | 49 | |
|  | 12 | B | **proliferation of muscle cells** | 3.66E-03 | CASZ1, CAV1, CXCL16, CYP1B1, GPER1, HDAC2, HEY1, HIF1A, HSD11B2, IRF1, KLF10, LAMA1, MEIS1, **MET,** MIR17HG, MMP1, NFAT5, NPR3, PDGFRB, PLAT, PLAUR, PLCE1, PRRX1, PTGS2, ROCK1, SOCS3, STAT1, STIM1, TXN, VCAN | 47 | |
|  | 13 | B | **proliferation of kidney cell lines** | 4.30E-03 | CAV2, CD24, CES2, ERBB4, FBN2, FGFR3, INPP5D, **MET,** NTN1, OXTR, PLAUR, PLCE1, PTGS2, RET, SOCS3, ZBTB5 | 56 | |
|  | 14 | D | **growth of neurites** | 1.90E-03 | AATK, ADCYAP1, CAV1, CDH2, DCLK1, DVL1, EGR1, ERBB4, FGFR3, FZD3, GDNF, GPM6A, ITGA1, ITGA4, KIF3C, LAMA1, MARCKS, **MET,** MIR17HG, NEDD4L, NRXN1, NTN1, PALLD, PDGFRB, PIK3CA, PLAT, POU4F1, RET, RIMS3, ROCK1, SCN1B, SOCS3, SRGAP2, TXN, UBE4B, VAPA, VCAN, VGF, WNT3A | 30 | |
|  | 15 | C | proliferation of T lymphocytes | 1.30E-03 | ABCG1, ADCYAP1, ANXA1, ARHGDIB, BLM, CAV1, CBX2, CD24, CD276, CD70, CD83, CSF2RA, CXCR4, CYLD, E2F2, EGR1, EGR2, EOMES, FTL, GAD1, HSPA1A/HSPA1B, ICOSLG/LOC102723996, IDO1, IL27RA, INPP5D, IRF1, JAG1, MAP2K6, MIR17HG, NFAT5, PIK3CA, PIM1, PPAT, PPP3CA, PRKD1, PTGS2, RCAN1, RHOH, SATB1, SOCS3, STAT1, STK17B, TCF7, TLR3, TNFRSF14, TXN, UBE2N, VDR, WNT3A | 9 | |
|  | 16 | C | proliferation of lymphocytes | 2.60E-03 | ABCG1, ADCYAP1, ANXA1, ARHGDIB, BLM, CAV1, CBX2, CD24, CD276, CD70, CD83, CSF2RA, CXCR4, CYLD, E2F2, EGR1, EGR2, EOMES, ERCC1, FTL, GAD1, HIF1A, HSPA1A/HSPA1B, ICOSLG/LOC102723996, IDO1, IKZF2, IL27RA, INPP5D, IRF1, JAG1, KLF3, MAP2K6, MEF2C, MIR17HG, NFAT5, PIK3CA, PIM1, PPAT, PPP3CA, PRKD1, PTGS2, RCAN1, RHOH, SATB1, SOCS3, STAT1, STK17B, TAC1, TCF7, TLR3, TNFRSF14, TXN, TYRP1, UBE2N, VDR, WNT3A | 16 | |
|  | 17 | B | proliferation of chondrocytes | 3.80E-03 | ERRFI1, FGFR3, HIF1A, IGFBP6, MAP2K6, PBX1, RBL1, RECK, STAT1 | 5 | |
|  | 18 | D | formation of red blood cells | 4.98E-04 | CD70, EPOR, HIF1A, PBX1, STAT1 | 73 | |
|  | 19 | D | formation of cells | 8.64E-04 | ADAM19, ADCYAP1, AKAP12, ARHGAP22, ARHGDIB, B4GALNT1, B4GALT1, CD70, CD83, CEP131, CLDN11, CXCR4, CYP1B1, DEAF1, DKK2, DLK1, EGR1, EGR2, EOMES, EPOR, ERBB4, ERCC1, EXPH5, FGF20, FOXP2, GDNF, GREM1, GSN, HEY1, HIF1A, INPP5D, IRF1, JAG1, JPH2, KIF1A, KLF10, LAMA1, LDB3, MAP2K6, MARCKSL1, MCOLN3, MEF2C, NR6A1, PALLD, PAX6, PBX1, PDGFA, PDGFRB, PHOX2B, PIM1, PTGS2, PTH1R, RDH10, REC8, RECK, ROM1, RPGR, SATB1, SEPP1, SNX10, SOCS3, SOX9, SPAG16, STAT1, STIM1, STYX, TCF7, TMSB10/TMSB4X, TXN, VDR, WNT3A, ZNF385A, ZNF521 | 13 | |
|  | 20 | D | formation of hematopoietic progenitor cells | 2.09E-03 | CD83, CXCR4, EGR1, EGR2, EOMES, EPOR, HIF1A, IRF1, PBX1, PIM1, SATB1, STAT1, TCF7 | 3 | |
|  | 21 | D | formation of colony-forming erythroid cells | 2.21E-03 | EPOR, HIF1A, PBX1 | 4 | |
|  | 22 | D | formation of erythroid progenitor cells | 2.79E-03 | EPOR, HIF1A, PBX1, STAT1 | 18 | |
|  | 23 | D | formation of blood cells | 2.82E-03 | CD70, CD83, CXCR4, EGR1, EGR2, EOMES, EPOR, GREM1, HIF1A, IRF1, MEF2C, PBX1, PIM1, SATB1, STAT1, STIM1, TCF7, ZNF385A | 8 | |
|  | 24 | D | formation of neurons | 3.23E-03 | ADAM19, ADCYAP1, CXCR4, FGF20, HEY1, MCOLN3, PAX6, PHOX2B | 18 | |
|  | 25 | D | formation of connective tissue cells | 5.33E-03 | CYP1B1, DKK2, DLK1, EGR1, GSN, HEY1, INPP5D, MEF2C, PAX6, PTGS2, PTH1R, SNX10, SOCS3, SOX9, STAT1, TXN, WNT3A, ZNF521 | 6 | |
|  | 26 | A | clonogenicity of tumor cell lines | 1.52E-03 | ACTA2, ALDH1A3, EMILIN2, EPOR, PDLIM4, PML | 39 | |
| **Trait in Circos Plot: A - proliferation of tumor cells; B - proliferation of solid tissue cells; C - proliferation of hematopoetic cells; D - various cell formation** | | | | | | | |
