# Supplementary material for: Chronic arsenic trioxide exposure leads to enhanced aggressiveness via Met oncogene addiction in cancer cells
Source: Oncotarget. 2016 Mar 28;7(19):27379–93. doi: 10.18632/oncotarget.8415 (PMC5053657; doi:10.18632/oncotarget.8415)
Supplement: Supplementary file 4 [file oncotarget-07-27379-s004.doc]

| **Table S4. GO Terms (biological processes) upregulated in A2780_ATO_ with a NOM p < 0.05** | | | | | | | | | | |  |
| --- | --- | --- | --- | --- | --- | --- | --- | --- | --- | --- | --- |
| **Nr.** | **NAME** | **GO Term ID:** | **Bio Process** | **SIZE** | **ES** | **NES** | **NOM p-val** | **FDR**  **q-val** | **FWER p-val** | Nr. | |
| 1 | SULFUR_METABOLIC_PROCESS | [GO:0006790](https://www.ebi.ac.uk/QuickGO/GTerm?id=GO:0006790) | 1 | 36 | 0.71 | 1.786 | **0.000** | 0.316 | 0.276 | 1516 | |
| 2 | HORMONE_METABOLIC_PROCESS | [GO:0042445](https://www.ebi.ac.uk/QuickGO/GTerm?id=GO:0042445) | 1 | 31 | 0.71 | 1.755 | **0.000** | 0.254 | 0.400 | 2388 | |
| 3 | CELLULAR_LIPID_METABOLIC_PROCESS | [GO:0044255](https://www.ebi.ac.uk/QuickGO/GTerm?id=GO:0044255) | 1 | 250 | 0.47 | 1.586 | **0.000** | 0.222 | 0.980 | 3697 | |
| 4 | LIPID_METABOLIC_PROCESS | [GO:0006629](https://www.ebi.ac.uk/QuickGO/GTerm?id=GO:0006629) | 1 | 320 | 0.43 | 1.492 | **0.000** | 0.310 | 0.999 | 3697 | |
| 5 | STEROID_METABOLIC_PROCESS | [GO:0008202](https://www.ebi.ac.uk/QuickGO/GTerm?id=GO:0008202) | 1 | 68 | 0.58 | 1.662 | **0.002** | 0.286 | 0.843 | 3626 | |
| 6 | CELLULAR_CARBOHYDRATE_METABOLIC_PROCESS | [GO:0044262](https://www.ebi.ac.uk/QuickGO/GTerm?id=GO:0044262) | 1 | 126 | 0.47 | 1.481 | **0.003** | 0.284 | 1 | 4068 | |
| 7 | CARBOHYDRATE_METABOLIC_PROCESS | [GO:0005975](https://www.ebi.ac.uk/QuickGO/GTerm?id=GO:0005975) | 1 | 179 | 0.44 | 1.466 | **0.003** | 0.298 | 1 | 4068 | |
| 8 | NEGATIVE_REGULATION_OF_CELLULAR_PROTEIN_METABOLIC_PROCESS | [GO:0032269](https://www.ebi.ac.uk/QuickGO/GTerm?id=GO:0032269) | 1 | 45 | 0.60 | 1.604 | **0.005** | 0.265 | 0.962 | 989 | |
| 9 | NEGATIVE_REGULATION_OF_PROTEIN_METABOLIC_PROCESS | [GO:0051248](https://www.ebi.ac.uk/QuickGO/GTerm?id=GO:0051248) | 1 | 48 | 0.60 | 1.630 | **0.007** | 0.244 | 0.918 | 989 | |
| 10 | AMINO_SUGAR_METABOLIC_PROCESS | [GO:0006040](https://www.ebi.ac.uk/QuickGO/GTerm?id=GO:0006040) | 1 | 19 | 0.72 | 1.619 | **0.014** | 0.248 | 0.938 | 1516 | |
| 11 | REGULATION_OF_PROTEIN_METABOLIC_PROCESS | [GO:0051246](https://www.ebi.ac.uk/QuickGO/GTerm?id=GO:0051246) | 1 | 173 | 0.41 | 1.334 | **0.017** | 0.408 | 1 | 3308 | |
| 12 | FATTY_ACID_METABOLIC_PROCESS | [GO:0006631](https://www.ebi.ac.uk/QuickGO/GTerm?id=GO:0006631) | 1 | 61 | 0.54 | 1.498 | **0.017** | 0.321 | 0.998 | 3458 | |
| 13 | REGULATION_OF_CELLULAR_PROTEIN_METABOLIC_PROCESS | [GO:0032268](https://www.ebi.ac.uk/QuickGO/GTerm?id=GO:0032268) | 1 | 162 | 0.42 | 1.356 | **0.028** | 0.402 | 1 | 2184 | |
| 14 | PROTEOGLYCAN_METABOLIC_PROCESS | [GO:0006029](https://www.ebi.ac.uk/QuickGO/GTerm?id=GO:0006029) | 1 | 21 | 0.69 | 1.597 | **0.036** | 0.262 | 0.968 | 2323 | |
| 15 | CYTOKINE_METABOLIC_PROCESS | [GO:0042107](https://www.ebi.ac.uk/QuickGO/GTerm?id=GO:0042107) | 1 | 42 | 0.55 | 1.443 | **0.037** | 0.320 | 1 | 563 | |
| 16 | COFACTOR_METABOLIC_PROCESS | [GO:0051186](https://www.ebi.ac.uk/QuickGO/GTerm?id=GO:0051186) | 1 | 51 | 0.54 | 1.459 | **0.042** | 0.296 | 1 | 3094 | |
| 17 | MONOCARBOXYLIC_ACID_METABOLIC_PROCESS | [GO:0032787](https://www.ebi.ac.uk/QuickGO/GTerm?id=GO:0032787) | 1 | 86 | 0.45 | 1.328 | **0.042** | 0.393 | 1 | 3559 | |
| 18 | ALCOHOL_METABOLIC_PROCESS | [GO:0006066](https://www.ebi.ac.uk/QuickGO/GTerm?id=GO:0006066) | 1 | 88 | 0.47 | 1.409 | **0.043** | 0.368 | 1 | 3626 | |
| 19 | CELLULAR_BIOSYNTHETIC_PROCESS | [GO:0044249](https://www.ebi.ac.uk/QuickGO/GTerm?id=GO:0044249) | 2 | 318 | 0.44 | 1.526 | **0.000** | 0.290 | 0.998 | 4023 | |
| 20 | BIOSYNTHETIC_PROCESS | [GO:0009058](https://www.ebi.ac.uk/QuickGO/GTerm?id=GO:0009058) | 2 | 466 | 0.40 | 1.415 | **0.000** | 0.366 | 1 | 4023 | |
| 21 | SULFUR_COMPOUND_BIOSYNTHETIC_PROCESS | [GO:0044272](https://www.ebi.ac.uk/QuickGO/GTerm?id=GO:0044272) | 2 | 18 | 0.77 | 1.694 | **0.006** | 0.291 | 0.691 | 1516 | |
| 22 | STEROID_BIOSYNTHETIC_PROCESS | [GO:0006694](https://www.ebi.ac.uk/QuickGO/GTerm?id=GO:0006694) | 2 | 23 | 0.72 | 1.673 | **0.007** | 0.302 | 0.803 | 2682 | |
| 23 | LIPID_BIOSYNTHETIC_PROCESS | [GO:0008610](https://www.ebi.ac.uk/QuickGO/GTerm?id=GO:0008610) | 2 | 97 | 0.49 | 1.487 | **0.008** | 0.299 | 1 | 3697 | |
| 24 | COFACTOR_BIOSYNTHETIC_PROCESS | [GO:0051188](https://www.ebi.ac.uk/QuickGO/GTerm?id=GO:0051188) | 2 | 21 | 0.70 | 1.635 | **0.013** | 0.256 | 0.905 | 2964 | |
| 25 | CARBOHYDRATE_BIOSYNTHETIC_PROCESS | [GO:0016051](https://www.ebi.ac.uk/QuickGO/GTerm?id=GO:0016051) | 2 | 49 | 0.59 | 1.590 | **0.014** | 0.229 | 0.976 | 4068 | |
| 26 | MACROMOLECULE_BIOSYNTHETIC_PROCESS | [GO:0009059](https://www.ebi.ac.uk/QuickGO/GTerm?id=GO:0009059) | 2 | 318 | 0.38 | 1.300 | **0.018** | 0.419 | 1 | 4068 | |
| 27 | TRANSLATION | [GO:0006412](https://www.ebi.ac.uk/QuickGO/GTerm?id=GO:0006412) | 2 | 178 | 0.40 | 1.320 | **0.024** | 0.399 | 1 | 5431 | |
| 28 | FATTY_ACID_OXIDATION | [GO:0019395](https://www.ebi.ac.uk/QuickGO/GTerm?id=GO:0019395) | 3 | 18 | 0.77 | 1.709 | **0.007** | 0.327 | 0.628 | 3458 | |
| 29 | CELLULAR_LIPID_CATABOLIC_PROCESS | [GO:0044242](https://www.ebi.ac.uk/QuickGO/GTerm?id=GO:0044242) | 3 | 34 | 0.62 | 1.597 | **0.018** | 0.245 | 0.968 | 3684 | |
| 30 | LIPID_CATABOLIC_PROCESS | [GO:0016042](https://www.ebi.ac.uk/QuickGO/GTerm?id=GO:0016042) | 3 | 37 | 0.61 | 1.592 | **0.023** | 0.238 | 0.975 | 3684 | |
| 31 | REGULATION_OF_MITOTIC_CELL_CYCLE | [GO:0007346](https://www.ebi.ac.uk/QuickGO/GTerm?id=GO:0007346) | 4 | 23 | 0.64 | 1.540 | **0.039** | 0.284 | 1 | 2026 | |
| **Biological processes classified as: 1 - metabolic, 2 - biosynthetic, 3 - lipid catabolism and 4 - reg. of mitotic cell cycle** | | | | | | | | | | | |
